# Morpho-Molecular Characterization of Microfungi Associated with *Phyllostachys* (Poaceae) in Sichuan, China

**DOI:** 10.3390/jof8070702

**Published:** 2022-07-01

**Authors:** Qian Zeng, Yi-Cong Lv, Xiu-Lan Xu, Yu Deng, Fei-Hu Wang, Si-Yi Liu, Li-Juan Liu, Chun-Lin Yang, Ying-Gao Liu

**Affiliations:** 1 College of Forestry, Sichuan Agricultural University, Chengdu 611130, China; zq1573037145@163.com (Q.Z.); yiconglv0616@163.com (Y.-C.L.); xuxiulanxxl@126.com (X.-L.X.); dy17711433642@126.com (Y.D.); scbzwfh@163.com (F.-H.W.); sharonwinchester@126.com (S.-Y.L.); liulijuan429@163.com (L.-J.L.); 2 Forestry Research Institute, Chengdu Academy of Agricultural and Forestry Sciences, Chengdu 611130, China

**Keywords:** bambusicolous fungi, molecular phylogeny, one new genus, systematics, two new species

## Abstract

In the present study, we surveyed the ascomycetes from bamboo of *Phyllostachys* across Sichuan Province, China. A biphasic approach based on morphological characteristics and multigene phylogeny confirmed seven species, including one new genus, two new species, and five new host record species. A novel genus *Paralloneottiosporina* is introduced to accommodate *Pa. sichuanensis* that was collected from leaves of *Phyllostachys violascens*. Moreover, the newly introduced species *Bifusisporella sichuanensis* was isolated from leaves of *P. edulis*, and five species were newly recorded on bamboos, four species belonging to *Apiospora*, viz. *Ap. yunnana*, *Ap. neosubglobosa*, *Ap. jiangxiensis*, and *Ap. hydei*, and the last species, *Seriascoma yunnanense,* isolated from dead culms of *P. heterocycla*. Morphologically similar and phylogenetically related taxa were compared. Comprehensive descriptions, color photo plates of micromorphology are provided.

## 1. Introduction

Bamboo is currently classified in the subfamily Bambusoideae of the extensive grass family Poaceae, and distributed worldwide. It comprises circa 1000 to 1500 species in up to 90 genera [1] and more than 70 species in *Phyllostachys* (Bambusoideae, Poaceae) [2,3]. Most bamboos are distributed in Southeast Asia, with China as the distribution center [4]. There are about 21 species of *Phyllostachys* in Sichuan, including *Phyllostachys edulis* (Carriere) J. Houzea, *P. heteroclada* Oliver, and *P. violascens* ‘Prevernalis’ S.Y. Chen et C.Y. Yao. Bamboos of *Phyllostachys* play an important role in native economy and ecology. They are used in furniture, and construction (e.g., fishing rods, flutes, flooring materials, chairs.) [5,6]. Bamboo shoots are used as food for humans and animals such as pandas [7,8]. In addition, it is an important ornamental plant for the landscape in China because of its evergreen and graceful appearance [9].

A review of the literature on bamboo-associated fungi reveals that nearly 1500 species have been described or recorded worldwide [10], including economically important pathogenic fungi, and a large number of saprobic and endophytic fungi [1,11,12,13]. Most bambusicolous fungi have been reported from Asia, especially Japan and Thailand, a few known from India and South America [1,12,14,15,16,17,18]. However, few studies have investigated the diversity and phylogeny on bamboo in China. The taxonomic studies on bambusicolous fungi are of great significance [19,20,21]. According to the literature review, about 85 species associated with *Phyllostachys* have been recorded. Teng [22] first reported the fungus *Oedocephalum glomerulosum* (Bull.) Sacc. on *Phyllostachys* in 1932. Tai listed 36 species of *Phyllostachys* from bamboo based on the reports on Chinese fungal resource until 1973 [23]. Chen investigated the phytogeography of forest fungi in China, North America, and Siberia, from which 33 species were found associated with *Phyllostachys* [24]. However, most of those identifications were conducted lacking molecular data and detailed micromorphology, and as most bamboos are unidentified, the relationship of bambusicolous fungi with bamboo species is not clear.

Due to the high fungal diversity on *Phyllostachys*, an ongoing investigation was conducted in several main producing or planting areas of bamboo *Phyllostachys* in Sichuan Province, China, including Ya’an City, Qionglai City, Chengdu City, and Yibin City. In this study, we provide detailed taxonomic features combining morphology and phylogeny on the fungi associated with *Phyllostachys* from Sichuan Province, China, which is a fundamental task for the bioresource collection on bambusicolous fungi.

## 2. Materials and Methods

### 2.1. Specimen Collection and Morphological Study

From 2020 to 2021, the specimens were collected from leaves, branches, and culms. The samples were kept in plastic bags and taken back to the laboratory after being photographed with a Sony DSC-HX3 digital camera. The fungi were isolated into pure culture based on single spore isolation [25]. Glass slide specimens were prepared by free-hand slicing with double-sided blades for morphologic observation. Morphological characteristics of ascomata and sporodochia were observed using a dissecting microscope, the NVT-GG (Shanghai Advanced Photoelectric Technology Co. Ltd., Shanghai, China), and photographed with a VS-800C micro-digital camera (Shenzhen Weishen Times Technology Co. Ltd., Shenzhen, China). An Olympus BX43 compound microscope with an Olympus DP22 digital camera was used to observe and photograph the microstructure of asci, ascospores, conidiophores, and conidia. Measurements were performed using Tarosoft^®^ Image Frame Work v.0.9.7 (Tarosoft (R), Nontha Buri, Thailand). Specimens were deposited at the Herbarium of Sichuan Agricultural University, Chengdu, China (SICAU), and pure cultures were deposited at the Culture Collection in Sichuan Agricultural University (SICAUCC).

### 2.2. DNA Extraction, PCR Amplification, and Nucleotide Sequencing

Genomic DNA was extracted from fresh mycelia which was cultured on PDA at 25 °C for 15–30 days, using a Trelief^TM^ Plant Genomic DNA Kit. Primers ITS5/ITS4 [26], NS1/NS4 [26], LR0R/LR5 [27], T1/Bt2b [28,29], RPB1-Ac/RPB1-Cr [30,31], and fRPB2-5F/fRPB2-7cR [32] were used for the amplification of internal transcribed spacers (ITS), the partial small subunit nuclear rDNA (SSU), the partial large subunit nuclear rDNA (LSU), the β-tubulin gene (*tub2*), the large subunit of RNA polymerase I (*rpb1*), and RNA polymerase II second largest subunit (*rpb2*) genes, respectively. Primers EF1-983F/EF1-2218R [33] and EF1-728F/EF2 [34,35] were employed for translation elongation factor 1-alpha (*tef1-α*) genes.

Amplification reactions were performed in 25 µL of total reaction that contained 22 µL Master Mix (Beijing TsingKe Biotech Co., Ltd., Beijing, China), 1 µL each of forward and reverse (10 µM) primers and 1 µL of DNA template. The amplification reactions were performed as described by Dai et al. [16] and Wang et al. [36]. PCR products were purified and sequenced at TsingKe Biological Technology Co., Ltd. (Chengdu, China). The resulting sequences were submitted to GenBank.

### 2.3. Sequence Alignment and Phylogenetic Analyses

Based on blast searches in GenBank, using ITS, LSU, SSU, *tef1-α*, *tub2*, *rpb1,* or *rpb2* sequence data, separate phylogenetic analyses were carried out to determine the placements of each fungal group (Table 1). Sequences for phylogenetic analyses were selected mainly from recently published literature and phylogenetic related sequences based on BLAST searches in GenBank (Table A1). Datasets were aligned using MAFFT v.7.407 [37], and ambiguous regions were excluded with BioEdit version 7.0.5.3 [38]. Maximum likelihood (ML) and Bayesian inference (BI) were constructed as described in Xu et al. [39]. The phylogram was visualized with FigureTree v. 1.4.3 and edited using Adobe Illustrator CS6 (Adobe Systems Inc., San Jose, CA, USA).

## 3. Results

### 3.1. Phylogenetic Analyses

**Figure 1:**
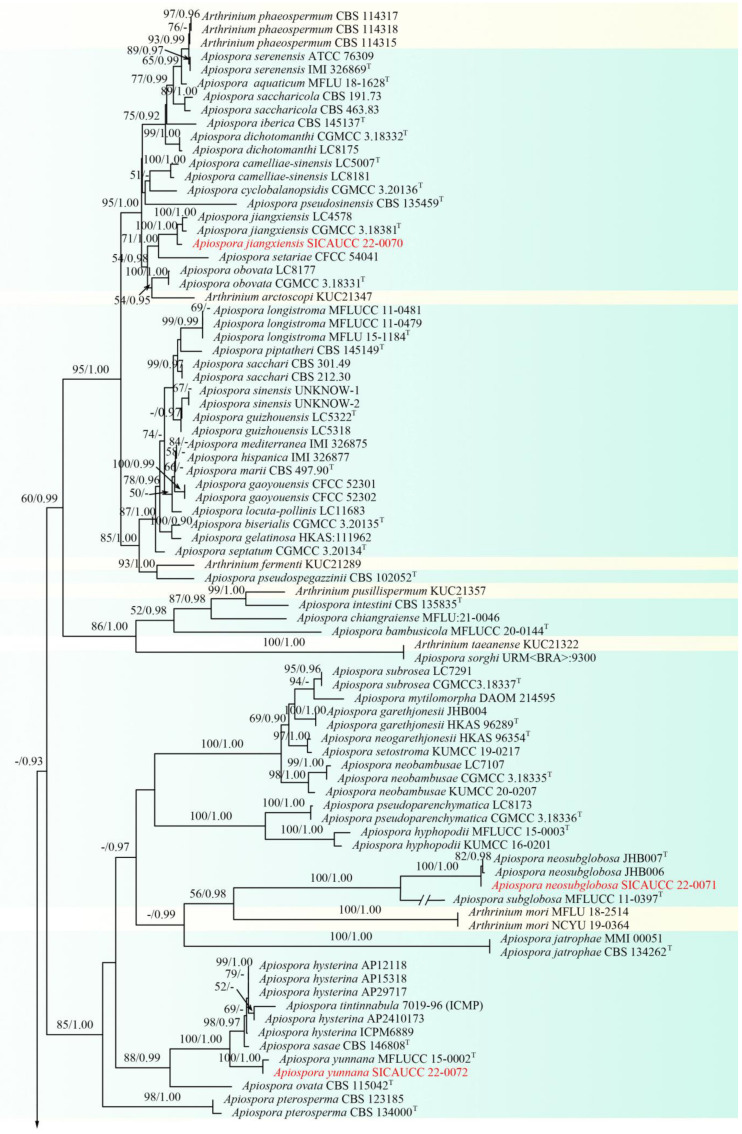

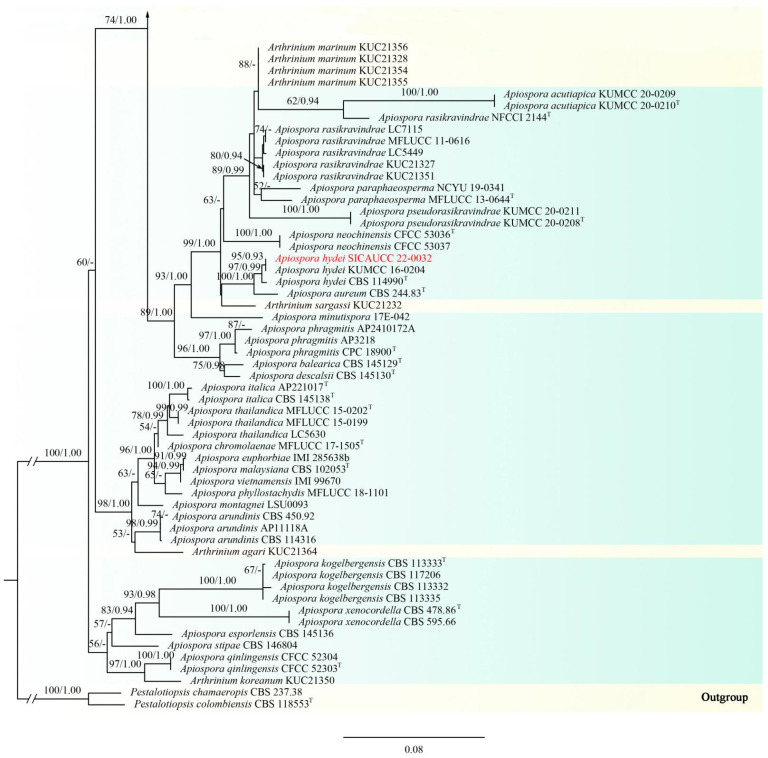
Phylogram generated from RAxML analysis based on combined ITS, LSU, *tub2,* and *tef1-α* sequence data of Apiosporaceae. Bootstrap support values for maximum likelihood (ML, left) higher than 50% and Bayesian posterior probabilities (BYPP, right) equal to or greater than 0.90 are indicated at the nodes, respectively. The sequences from ex-type strains are marked by a superscript symbol T. The newly generated sequences are written in red. *Arthrinium* species with yellow background were temporarily not combined to *Apiospora*.

A combined dataset (ITS, LSU, *tef1-α*, *tub2*) comprising 138 taxa within Apiosporaceae, which is rooted with *Pestalotiopsis chamaeropis* (CBS 237.38) and *Pe. colombiensis* (CBS 118553) (Pestalotiopsidaceae, Amphisphaeriales), was used for the phylogenetic analyses. The alignment contained 5875 characters (ITS = 999, LSU = 1382, *tef1-α* = 1651, *tub2* = 1844), including gaps. The best scoring RAxML tree with a final likelihood value of −36198.939448 is presented. The matrix had 2337 distinct alignment patterns, with 64.85% of undetermined characters or gaps. Estimated base frequencies were as follows: A = 0.237208, C = 0.257370, G = 0.253511, T = 0.251911, with substitution rates AC = 1.104968, AG = 2.746651, AT = 1.143208, CG = 0.910079, CT = 4.335389, GT = 1.000000. The gamma distribution shape parameter α = 0.269105, and the tree length = 3.509694. In the phylogenetic trees generated from ML and BI analyses, the strain SICAUCC 22-0032 clustered with the known species *Apiospora hydei* (KUMCC 16-0204, CBS 114990) in a clade with 97% ML and 0.99 BYPP support value, strain SICAUCC 22-0070 clustered with *Ap. jiangxiensis* (CGMCC 3.18381, LC4578) with high support values (100% ML and 1.00 BYPP), strain SICAUCC 22-0071 clustered with *Ap. neosubglobosa* (JHB006, JHB007) in a clade with 100% ML and 1.00 BYPP support value, and strain SICAUCC 22-0072 clustered with the *Ap. yunnana* (MFLUCC 15-0002) in a clade with 100% ML and 1.00 BYPP support values (Figure 1).

**Figure 2:**
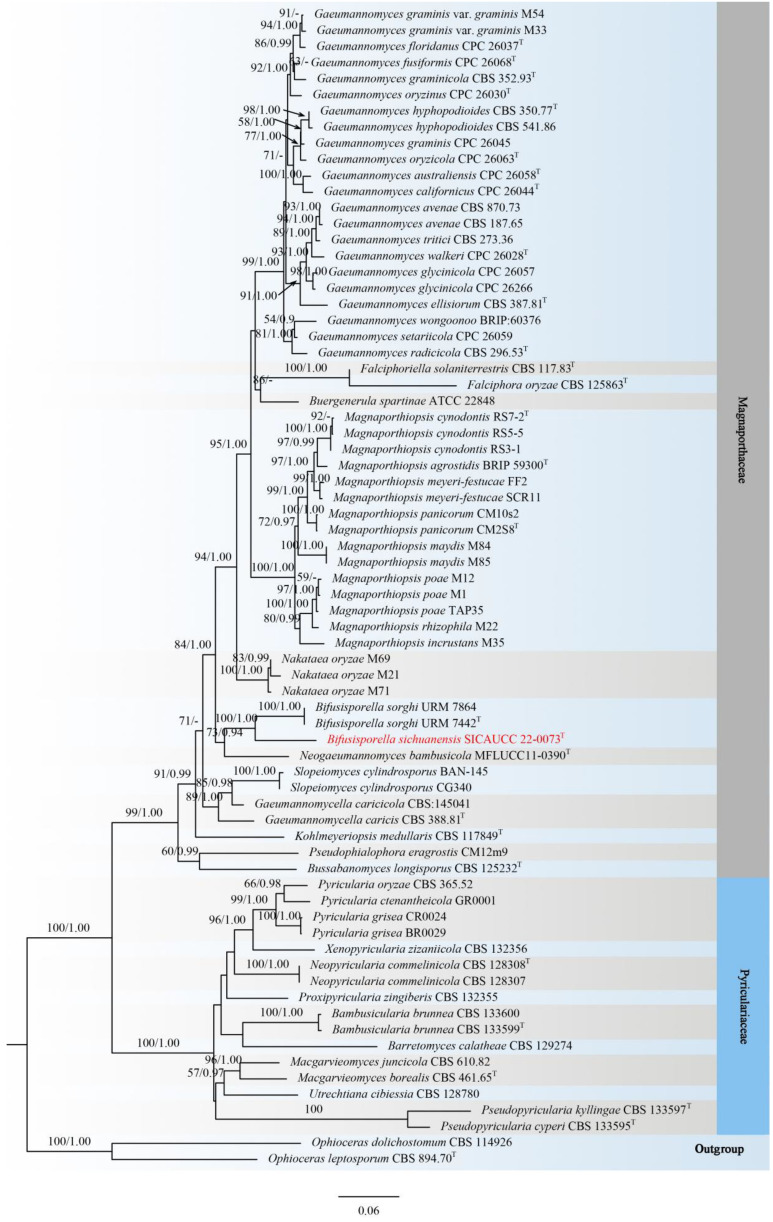
Phylogram generated from RAxML analysis based on combined ITS, LSU, *rpb1,* and *tef1-α* sequence data of Magnaporthaceae and Pyriculariaceae. Bootstrap support values for maximum likelihood (ML, left) higher than 50% and Bayesian posterior probabilities (BYPP, right) equal to or greater than 0.90 are indicated at the nodes, respectively. The sequences from ex-type strains are marked by a superscript symbol T. The newly generated sequence is written in red.

Phylogenetic analyses of a concatenated aligned dataset (ITS, LSU, *rpb1*, *tef1-α*), including 70 taxa within Magnaporthaceae and Pyriculariaceae, were conducted and rooted with *Ophioceras dolichostomum* (CBS 114926) and *O. leptosporum* (CBS 894.70) (Ophioceraceae, Magnaporthales). The alignment contained 4094 characters (ITS = 899, LSU = 1105, *rpb1* = 1047, *tef1-α* = 1043), including gaps. The best scoring RAxML tree with a final likelihood value of −31022.648763 is presented. The matrix had 1923 distinct alignment patterns, with 36.77% of undetermined characters or gaps. Estimated base frequencies were as follows: A = 0.243596, C = 0.275654, G = 0.281915, T = 0.198836, with substitution rates AC = 1.103727, AG = 2.292134, AT = 1.431191, CG = 0.918700, CT = 5.773674, GT = 1.000000. The gamma distribution shape parameter α = 0.319184, and the tree length = 3.313974. In the phylogenetic tree (Figure 2), the novel species *Bifusisporella sichuanensis* constitutes a highly supported independent lineage (ML = 100%, BYPP = 1.00) with *B. sorghi* (URM 7864, URM 7442).

**Figure 3:**
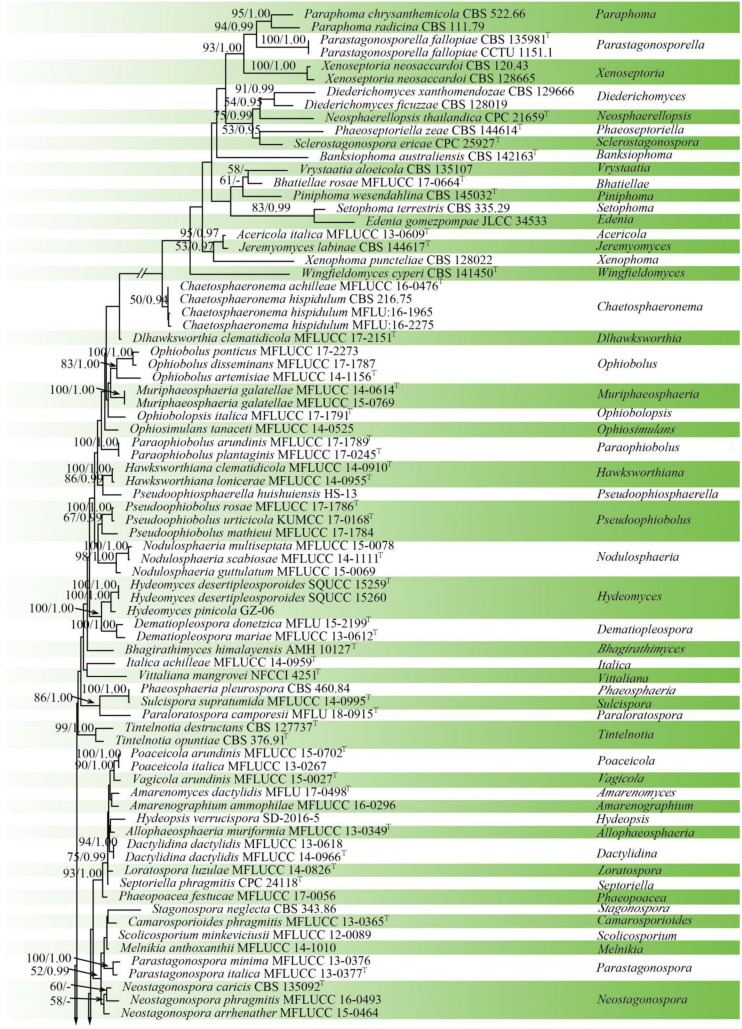

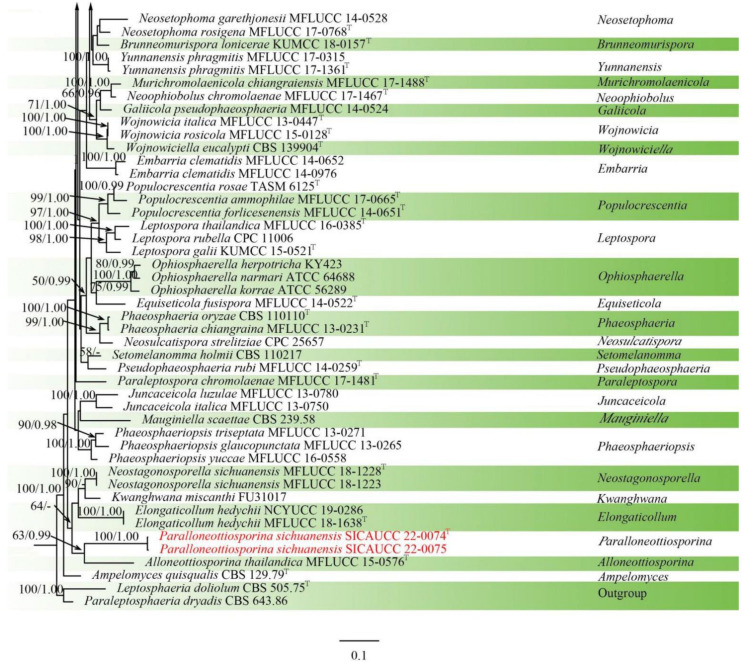
Phylogram generated from RAxML analysis based on combined ITS, LSU, SSU, and *tef1-α* sequence data of Phaeosphaeriaceae. Bootstrap support values for maximum likelihood (ML, left) higher than 50% and Bayesian posterior probabilities (BYPP, right) equal to or greater than 0.90 are indicated at the nodes, respectively. The sequences from ex-type strains are marked by a superscript symbol T. The newly generated sequences are written in red.

The concatenated aligned dataset of ITS, LSU, SSU, *tef1-α* sequences, including 124 ingroup taxa within Phaeosphaeriaceae and two outgroup taxa in Leptosphaeriaceae, were used for the phylogenetic analyses of *Paralloneottiosporina*. The alignment contained 5851 characters (ITS = 1469, LSU = 1433, SSU = 1548, *tef1-α* = 1401), including gaps. The best scoring RAxML tree with a final likelihood value of −46908.078740 is presented. The matrix had 2382 distinct alignment patterns, with 55.68% of undetermined characters or gaps. Estimated base frequencies were as follows: A = 0.246158, C = 0.236637, G = 0.264322, T = 0.252883, with substitution rates AC = 1.087661, AG = 2.657942, AT = 2.045792, CG = 0.863381, CT = 6.106747, GT = 1.000000. The gamma distribution shape parameter α = 0.263651, and the tree length = 7.503091. In the phylogenetic tree generated from ML and BI analyses, the novel species *Paralloneottiosporina sichuanensis* (SICAUCC 22-0074, SICAUCC 22-0075) constitutes a moderately supported independent lineage (63% ML/0.99 BYPP statistical support) with the species *Alloneottiosporina thailandica* (MFLUCC 15-0576) (Figure 3).

**Figure 4:**
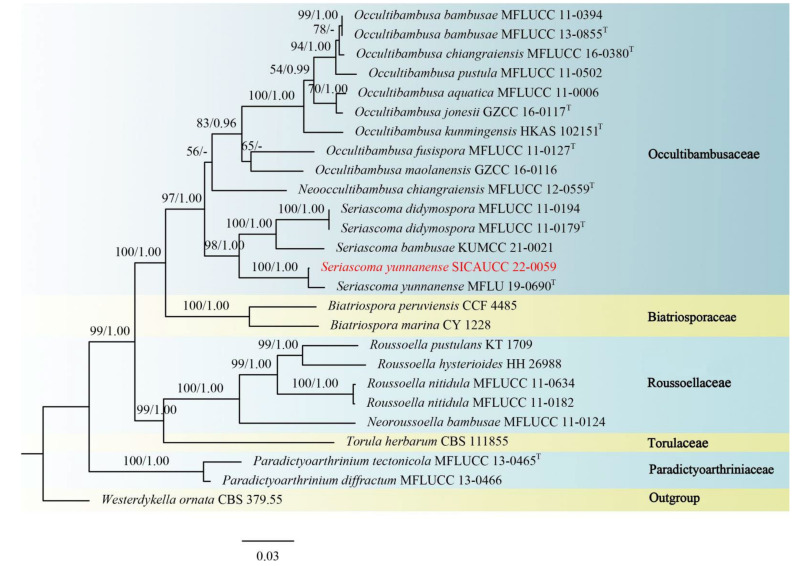
Phylogram generated from RAxML analysis based on combined ITS, LSU, *rpb2,* and *tef1-α* sequence data of isolates within Bambusicolaceae and other representative species in Biatriosporaceae, Roussoellaceae, Torulaceae, and Paradictyoarthriniaceae. Bootstrap support values for maximum likelihood (ML, left) higher than 50% and Bayesian posterior probabilities (BYPP, right) equal to or greater than 0.90 are indicated at the nodes, respectively. The sequences from ex-type strains are marked by a superscript symbol T. The newly generated sequence is written in red.

A combined dataset (ITS, LSU, SSU, *tef1-α*, *rpb2*) comprising 25 taxa within Bambusicolaceae, Biatriosporaceae, Roussoellaceae, Torulaceae, and Paradictyoarthriniaceae was used for phylogenetic analyses of *Seriascoma*, and the *Westerdykella ornata* (CBS 379.55) (Sporormiaceae) was used as outgroup taxon. The alignment contained 6569 characters (LSU = 1383, SSU = 1741, *tef1-α* = 1346, *rpb2* = 2099), including gaps. The best scoring RAxML tree with a final likelihood value of −22606.776997 is presented. The matrix had 1406 distinct alignment patterns, with 48.40% of undetermined characters or gaps. Estimated base frequencies were as follows: A = 0.250203, C = 0.247742, G = 0.269455, T = 0.232600, with substitution rates AC = 1.348170, AG = 4.119625, AT = 1.278817, CG = 1.296090, CT = 9.080955, GT = 1.000000. The gamma distribution shape parameter α = 0.146142, and the tree length = 1.192279. According to the phylogenetic tree (Figure 4), the strain (SICAUCC 22-0059) clustered with *Seriascoma yunnanense* (MFLU 19-0690) in a clade with 100% ML and 1.00 BYPP statistical support.

### 3.2. Taxonomy

Apiosporaceae K.D. Hyde, J. Fröhl., Joanne E. Taylor & M.E. Barr, Sydowia. 50 (1): 23 (1998).

**Figure 5:**
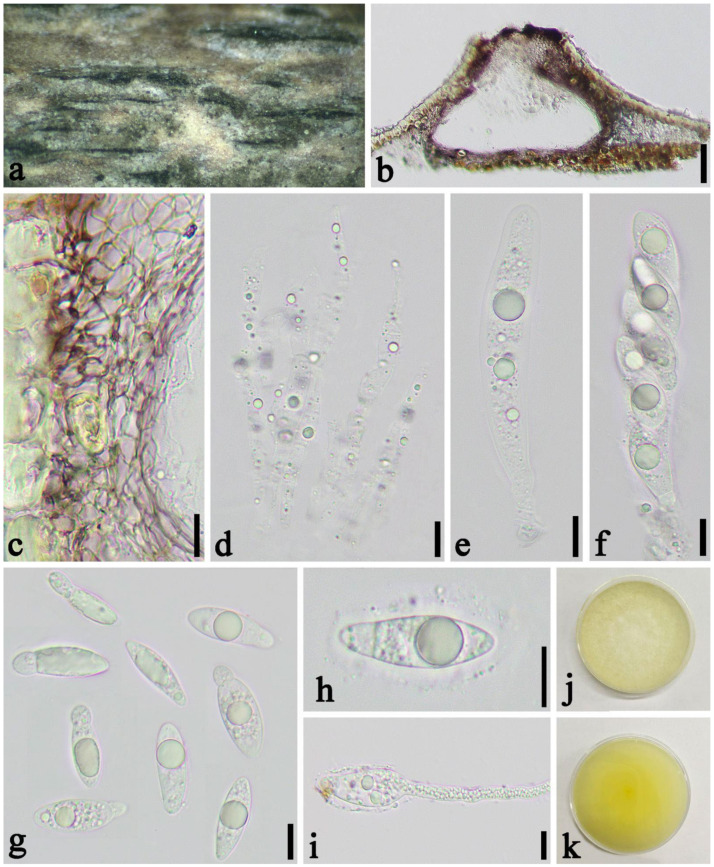
*Apiospora hydei* (SICAU 22-0032). (**a**) Ascostromata developing on bamboo branches. (**b**) Vertical sections of ascostromata. (**c**) Peridium. (**d**) Paraphyses. (**e**,**f**) Asci. (**g**,**h**) Ascospores. (**i**) Germinating ascospore. (**j**,**k**) Cultures on PDA. Scale bars: (**b**) = 50 μm, (**c**–**i**) = 10 μm.

*Apiospora hydei* (Crous) Pintos & P. Alvarado, Fungal Systematics and Evolution. 7: 206 (2021) (Figure 5).

≡ *Arthrinium hydei* Crous, IMA Fungus 4(1): 142 (2013).

Saprobic on dead culms of *Phyllostachys nigra* (Lodd. ex Lindl.) Munro. *Sexual morph*: *Ascostromata* 421–1343 × 174–387 × 176–245 μm ($\bar{x}$ = 705 × 267 × 198 μm, *n* = 30), solitary to gregarious, immersed, fusiform to ellipsoid, dark brown to black, multi-loculate, with long axis. *Peridium* 17–46 μm wide, composed of 8–15 layers of brown to hyaline cells of *textura angularis* to *prismatica*. *Hamathecium* 2–6.5 μm wide, composed of dense, long, septate, and unbranched paraphyses. *Asci* 81–123 × 16–23 μm, ($\bar{x}$ = 116 × 180 μm, *n* = 50), 8–spored, unitunicate, broadly cylindrical, slightly curved, with a short pedicel, apically rounded. *Ascospores* 24–30 × 7–11 μm, ($\bar{x}$ = 26 × 10 μm, *n* = 50), 2-seriate, elliptical, 1–septate, with a large, curved upper cell and small lower cell, with narrowly rounded ends, hyaline, guttules, smooth-walled, surrounded by gelatinous sheath. *Asexual morph*: see Crous et al. [40].

Material examined: China, Sichuan Province, Chengdu City, Wenjiang District (19°30′42.22″ N, 103°51′19″ E, Alt. 528 m), on dead culms of *Phyllostachys nigra*, 14 March 2021, Yi-cong Lv, LYC202103003 (SICAU 22-0032), living culture SICAUCC 22-0032.

Culture characters: Ascospores germinate within 24 h. Colonies grow fast on PDA, reaching 6 cm after one week at 25 °C, under 12 h light/12 h dark, and are cottony, circular, and white from above and light yellow below, with irregular edge.

Notes: *Apiospora hydei* was introduced based on the asexual morph characters and phylogeny analyses by Crous et al. [40]. Morphological comparisons were impossible due to the lack of sexual morph between our isolates and the ex-type strain (CBS 114990), but it is similar to *A. hydei* in sexual descriptions provided by Dai et al. [41]. Nucleotide comparisons of ITS, LSU, *tef1-α* and *tub2* (SICAUCC 22-0033) showed high homology with the sequences of *A. hydei* (CBS 114990), similarities are 100% (528/528, 0 gaps), 99.77% (896/898, 0 gaps), 99.71% (355/356, 0 gaps), and 98.82% (754/763, 0 gaps), respectively.

**Figure 6:**
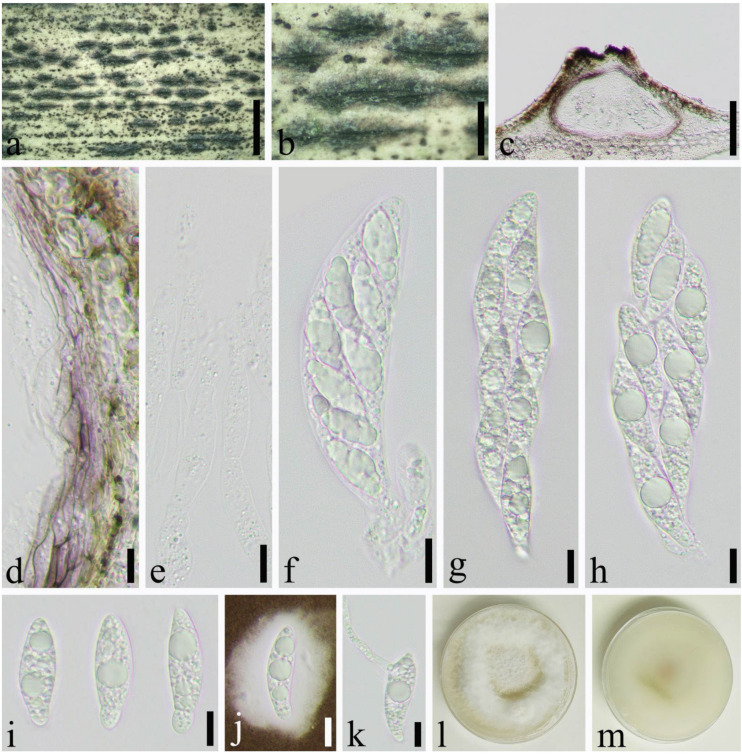
*Apiosporajiangxiensis* (SICAU 22-0070). (**a**,**b**) Ascostromata developing on bamboo culm. (**c**) Vertical sections of ascostromata. (**d**) Peridium. (**e**) Paraphyses. (**f**–**h**) Asci. (**i**,**j**) Ascospores. (**k**) Germinating ascospore. (**l**,**m**) Cultures on PDA. Scale bars: (**a**) = 2 mm, (**b**) = 500 μm, (**c**) = 100 μm, (**d**–**k**) = 10 μm.

*Apiospora jiangxiensis* (M. Wang & L. Cai) Pintos & P. Alvarado, Fungal Systematics and Evolution 7: 206 (2021) (Figure 6).

≡ *Arthrinium jiangxiense* M. Wang & L. Cai, in Wang, Tan, Liu & Cai, MycoKeys 34(1): 14 (2018).

Saprobic on dead culms of *Phyllostachys heteroclada* Oliver. *Sexual morph*: *Ascostromata* 575–1334 × 274–444 × 134–157 μm ($\bar{x}$ = 876 × 355 × 143 μm, *n* = 30), solitary to gregarious, multi-loculate, immersed, fusiform to ellipsoid, black, with long axis broken at the top. *Peridium* 9.0–44 μm wide ($\bar{x}$ = 21 μm, *n* = 25), composed of several layers of brown to hyaline cells of *textura angularis* to *prismatica*. *Hamathecium* 4.0–11 μm wide, composed of dense, long, septate, unbranched, paraphyses. *Asci* 83–114 × 18–28 μm ($\bar{x}$ = 104 × 23 μm, *n* = 50), 8–spored, unitunicate, broadly cylindrical to long clavate, with a short pedicel, slightly curved, apically rounded. *Ascospores* 32–37 × 9.6–11 μm ($\bar{x}$ = 34 × 10 μm, *n* = 50), 2–seriate, 1–septate, elliptical, with a large, curved, upper cell and small lower cell, with narrowly rounded ends, hyaline, smooth-walled, with many guttules, surrounded by gelatinous sheath attached. *Asexual morph*: see Wang et al. [36].

Material examined: China, Sichuan Province, Luzhou City, Xuyong District (27°53′28″ N, 105°16′36″ E, Alt. 1350 m), on dead culm of *Phyllostachys heteroclada*, 26 July 2021, Qian Zeng, ZQ202107133 (SICAU 22-0070), living culture SICAUCC 22-0070.

Culture characters: Ascospores germinate on PDA within 24 h. Colonies grow fast on PDA, reaching 6 cm after 1 week at 25 °C, under 12 h light/12 h dark, and are cottony, white, circular, with irregular edge.

Notes: Specimen in our study shared similar morphology with the original description of *Apiospora jiangxiensis* by Wang et al. [36]. Nucleotide comparisons of ITS, LSU, and *tub2* (SICAUCC 22-0070) showed high homology with the sequences of *Ap. jiangxiensis* (CGMCC 3.18381), similarities are 100% (541/541, 0 gaps), 99.09% (436/440, 0 gaps), and 98.22% (717/730, 0 gaps), respectively. However, the latter lack *tef1-α* sequences for further comparisons.

**Figure 7:**
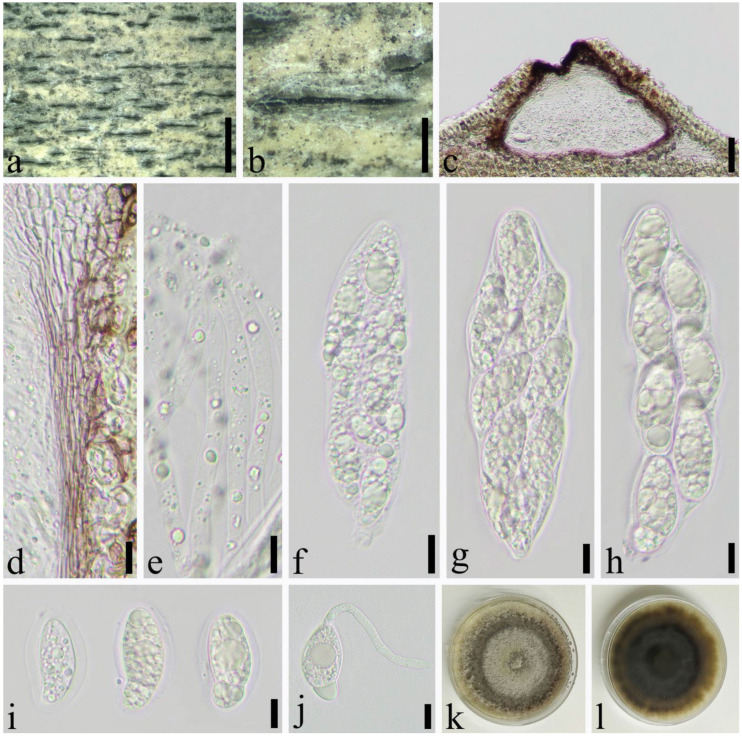
*Apiosporaneosubglobosa* (SICAU 22-0071). (**a**,**b**) Ascostromata developing on bamboo culm. (**c**) Vertical sections of ascostromata. (**d**) Peridium. (**e**) Paraphyses. (**f**–**h**) Asci. (**i**) Ascospores. (**j**) Germinating ascospore. (**k**,**l**) Cultures on PDA. Scale bars: (**a**) = 2 mm, (**b**) = 500 μm, (**c**) = 50 μm, (**d**–**j**) = 10 μm.

*Apiospora neosubglobosa* (D.Q. Dai & H.B. Jiang) Pintos & P. Alvarado, Fungal Systematics and Evolution 7: 206 (2021) (Figure 7).

≡ *Arthrinium neosubglobosum* D.Q. Dai & H.B. Jiang, Mycosphere 7(9): 1337 (2017).

Saprobic on dead culms of *Phyllostachys bissetii* McClure. *Sexual morph*: *Ascostromata* 330–1092 × 198–354 × 134–224 μm ($\bar{x}$ = 632 × 250 × 174 μm, *n* = 30), gregarious, immersed, multi-loculate, fusiform to ellipsoid, dark brown to black, with long axis broken at the top. *Peridium* 17.0–46 μm wide ($\bar{x}$ = 19 μm, *n* = 25), composed of several layers of brown to hyaline, cells of *textura angularis* to *prismatica. Hamathecium* 3.5–6.0 μm wide, composed of dense, long, septate, unbranched, paraphyses. *Asci* 94–137 × 23–40 μm ($\bar{x}$ = 125 × 31 μm, *n* = 50), 8-spored, unitunicate, broadly cylindrical to long clavate, with a short pedicel, slightly curved, apically rounded. *Ascospores* 28–36 × 13–15 μm ($\bar{x}$ = 32 × 14 μm, *n* = 50), 2–seriate, 1–septate, elliptical, with a large, curved, upper cell and small lower cell, with narrowly rounded ends, hyaline, smooth-walled, with many guttules, surrounded by gelatinous sheath attached. *Asexual morph*: see Dai et al. [16].

Material examined: CHINA, Sichuan Province, Luzhou City, Xuyong District (27°52′5″ N, 105°16′23″ E, Alt. 1470 m), on dead culm of *Phyllostachys bissetii*, 26 July 2021, Qian Zeng, ZQ202107128 (SICAU 22-0071), living culture SICAUCC 22-0071.

Cultural characters: Ascospores germinate on PDA within 24 h. Colonies grow fast on PDA, reaching 4 cm after 1 week at 25 °C, under 12 h light/12 h dark, and are cottony, circular, initially white, then brown, with regular edge.

Notes: *Apiospora neosubglobosa* was described by Dai et al. based on the morphological characteristics and molecular phylogeny [16]. Strain SICAUCC 22-0071 clustered with ex–type strain (JHB007) with high bootstrap support (100% ML and 1.00 BYPP). Nucleotide comparisons of ITS and LSU (SICAUCC 22-0071) showed high homology with the sequences of *Ap. neosubglobosa* (JHB007), similarities are 99.84% (649/650, 0 gaps), 100% (1173/1173, 0 gaps), respectively.

**Figure 8:**
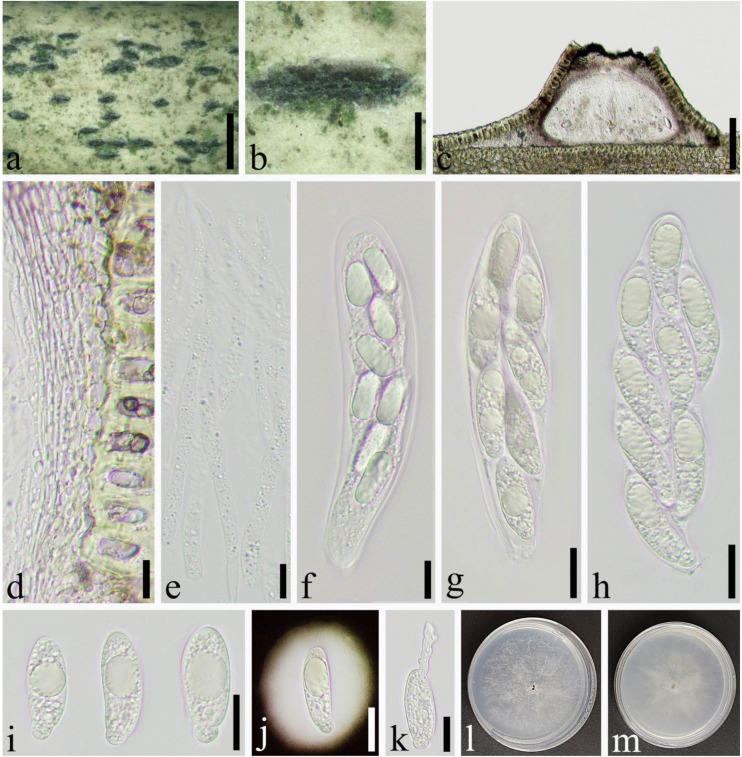
*Apiosporayunnana* (SICAU 22-0072). (**a**,**b**) Ascostromata developing on bamboo culm. (**c**) Vertical sections of ascostromata. (**d**) Peridium. (**e**) Paraphyses. (**f**–**h**) Asci. (**i**,**j**) Ascospores. (**k**) Germinating ascospore. (**l**,**m**) Cultures on PDA. Scale bars: (**a**) = 2 mm, (**b**) = 500 μm, (**c**) = 100 μm, (**d**–**f**) = 10 μm, (**g**–**k**) = 20 μm.

*Apiospora yunnana* (D.Q. Dai & K.D. Hyde) Pintos & P. Alvarado, Fungal Systematics and Evolution 7: 207 (2021) (Figure 8).

≡ *Arthrinium yunnanum* D.Q. Dai & K.D. Hyde, Fungal Diversity 82: 69 (2016).

Saprobic on culms of *Phyllostachys aurea* Carr. ex A. et C. Riv. *Sexual morph*: *Ascostromata* 624–1307 × 253–510 × 165–211 μm ($\bar{x}$ = 892 × 359 × 188 μm, *n* = 30), gregarious, multi-loculate, immersed, fusiform to ellipsoid, black, with long axis broken at the top. *Peridium* 8.5–43 μm wide ($\bar{x}$ = 17 μm, *n* = 25), composed of several layers of brown to hyaline cells of *textura angularis* to *prismatica*. *Hamathecium* 3.5–8.0 μm wide, composed of dense, long, septate, unbranched paraphyses. *Asci* 89–144 × 18–40 μm ($\bar{x}$ = 120 × 32 μm, *n* = 50), 8–spored, unitunicate, broadly cylindrical to long clavate, no pedicel, slightly curved, apically rounded. *Ascospores* 30–42 × 10–13 μm ($\bar{x}$ = 36 × 12 μm, *n* = 50), 2–seriate, 1–septate, elliptical, with a large, curved, upper cell and a small lower cell, with narrowly rounded ends, hyaline, smooth-walled, with many guttules, surrounded by gelatinous sheath attached. *Asexual morph*: see Dai et al. [16].

Material examined: China, Sichuan Province, Yibin City, Changning District (28°28′8″ N, 105°0′16″ E, Alt. 890 m), on dead culm of *Phyllostachys aurea*, 23 July 2021, Qian Zeng, ZQ202107027 (SICAU 22-0072), living culture, SICAUCC 22-0072.

Culture characters: Ascospores germinate on PDA within 24 h and germ tubes produced from sides. Colonies grow fast on PDA, reaching 6 cm after 1 week at 25 °C, under 12 h light/12 h dark, and are cottony, circular, and white with irregular edge.

Notes: The sexual and asexual morph of *Apiospora yunnana* was reported by Dai et al. [16]. Morphologically, our observations were identical to the sexual descriptions provided by Daiet et al. [16]. Nucleotide comparisons of ITS and LSU (SICAUCC 22-0072) showed high homology with the sequences of *Ap. yunnana* (MFLUCC 15-0002), similarities are 99.85% (667/668, 0 gaps), 100% (847/847, 0 gaps), respectively. However, the latter lack *tef1-α* and *tub2* sequences for further comparisons.

Magnaporthales Thongkantha, Vijaykrishna & K.D. Hyde. Fungal Diversity. 34: 157–173 (2009).

Magnaporthaceae P.F. Cannon, Systema Ascomycetum 13: 26 (1994).

*Bifusisporella* R.M.F. Silva, R.J.V. Oliveira, J.D.P. Bezerra, J.L. Bezerra, C.M. Souza-Motta & G.A. Silva, Mycological Progress 18(6): 852 (2019).

Type species: *Bifusisporella sorghi* R.M.F. Silva, R.J.V. Oliveira, J.D.P. Bezerra, J.L. Bezerra, C.M. Souza-Motta & G.A. Silva.

Description: Endophytic and parasitic fungi on Poaceae. *Sexual morph: Ascomata* separate or gregarious, subglobose, black, coriaceous, semi-immersed, unilocular or multilocular. *Peridium* with hyaline to brown cells of *textura angularis*. *Hamathecium* hyaline, with distinct septa, wider at the base, tapering towards the apex. *Asci* 8–spored, cylindrical, with a J-, apical ring, developing from the base and periphery of the ascomata, with a short pedicel. *Ascospores* biseriate, hyaline, fusiform, with distinct septa, with narrowly rounded ends, without appendages. *Asexual morph:* Found in *Bifusisporella sorghi* cultures by Silva et al. [42].

Notes: *Bifusisporella* was introduced as a new genus to accommodate *B. sorghi* based on morphology and phylogeny. At present, *Bifusisporella* comprises only the ex-type species *B. sorghi*, and no records on its sexual morph. The new species *B. sichuanensis* is well-supported within *Bifusisporella,* which suggests that there is a need to amend the morphological circumscriptions of the genus.

**Figure 9:**
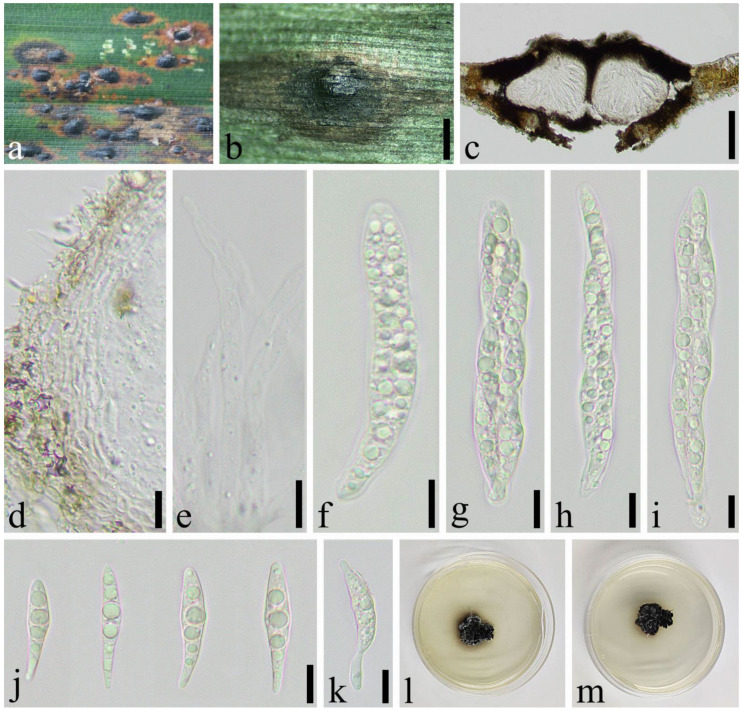
*Bifusisporella sichuanensis* (SICAU 22-0073). (**a**,**b**) Ascostromata developing on the host. (**c**) Vertical sections of ascostromata. (**d**) Peridium. (**e**) Pseudoparaphyses. (**f**–**i**) Asci. (**j**) Ascospores. (**k**) Germinating ascospore. (**l**,**m**) Cultures on PDA. Scale bars: (**b**) = 500 µm, (**c**) = 100 µm, (**d**–**k**) = 10 µm.

*Bifusisporella sichuanensis* Q. Zeng, Y.C. Lv & C.L. Yang, sp. nov. (Figure 9).

Index Fungorum: IF559625

Etymology: Refers to the region from where the fungus was collected.

Holotype: SICAU 22-0073

Parasitic on living leaves of *Phyllostachys* *edulis* (Carriere) J. Houzeau. *Sexual morph: Ascostromata* 536–1672 × 332–849 × 125–245 μm ($\bar{x}$ = 1103 × 591 × 193 μm, *n* = 30), separate or gregarious, subglobose, black, coriaceous, semi-immersed, unilocular or multilocular, glabrous. *Peridium* 14–34 μm wide ($\bar{x}$ = 20 μm, *n* = 30), composed of 3–9 layers, with hyaline to brown cells of *textura angularis*. *Hamathecium*, hyaline, cellular, with distinct septa. *Asci* 79–126 × 9.5–13 μm ($\bar{x}$ = 99 × 11 μm, *n* = 30), 8–spored, bitunicate, cylindrical, with an apical chamber and a short pedicel. *Ascospores* 22–35 × 5.0–6.5 μm ($\bar{x}$ = 29 × 5.5 μm, *n* = 50), overlapping, biseriate, hyaline, fusiform, 3–septate, rarely constricted at septate, with narrowly rounded ends, smooth-walled, guttules, without gelatinous sheath. *Asexual morph*: Undetermined.

Material examined: China, Sichuan Province, Yibin City, Xingwen District (28°15′22″ N, 105°6′29″ E, Alt. 850 m), on living to nearly dead leaves of *Phyllostachys edulis*, 25 July 2021, Qian Zeng, ZQ202107111 (SICAU 22-0073 holotype), ex-type living culture, SICAUCC 22-0073.

Culture characters: Ascospores germinate in sterilized water within 12 h at 25 °C. Colonies grow slow on PDA, reaching approximately 2 cm in 30 days at 25 °C, under 12 h light/12 h dark, and are irregular, black, frilly with white margin, and black on the back of colonies.

Notes: *Bifusisporella sichuanensis* is phylogenetically close (100% ML and 1.00 BYPP) to *B. sorghi* (URM 7442) introduced by Silva et al. [42], which is described with asexual morph. However, striking base-pair differences are noted, viz. 11.43% (55/481, 0 gaps), 3.36% (27/803, 0 gaps), 5.11% (24/469, 0 gaps), 9.04% (64/708, 0 gaps) in the ITS, LSU, *tef1-α* and *rpb1*, respectively. Hence, our collection is proposed as a new species.

Pleosporales Luttr. ex M.E. Barr, Prodromus to class Loculoascomycetes: 67 (1987).

Phaeosphaeriaceae M.E. Barr, Mycologia 71: 948 (1979).

*Paralloneottiosporina* Q. Zeng, Y.C. Lv & C.L. Yang, gen. nov.

Index Fungorum: IF559626.

Type species: *Paralloneottiosporina sichuanensis* Q. Zeng, Y.C. Lv & C.L. Yang.

Etymology: Name reflects the morphological similarity to the genus *Alloneottiosporina*.

Parasitic on living to nearly dead leaves of *Phyllostachys violascens* ‘Prevernalis’ S.Y. Chen et C.Y. Yao. *Sexual morph*: *Ascomata* visible as raised to superficial on host, gregarious, globose to subglobose or dome shape, dark brown to black, unilocular, glabrous. *Ostiole* single, circular, centrally located. *Peridium* multi-layered, brown to dark brown cells of *textura angularis*. *Hamathecium* hyaline, numerous, septate, often constricted at septa. *Asci* 8-spored, bitunicate, rounded at apex, cylindrical, curved, with a short pedicel. *Ascospores* hyaline, fusiform, 1–2 septate, constricted at the septum, guttules, smooth-walled, with narrowly rounded ends. *Asexual morph*: *Conidiomata* brown to dark brown, globose to long ellipsoid, coriaceous, semi-immersed, unilocular, gregarious, glabrous. *Conidiomatal wall* comprising multi-layered, dark brown to black cells of *textura angularis*. *Conidia* ellipsoid to ovoid, 1–septate, slightly constricted at the septum, smooth-walled, hyaline, with a rounded apex and a truncated base, guttules.

Notes: *Paralloneottiosporina* resembles *Alloneottiosporina* in asexual status having semi-immersed, unilocular, gregarious, glabrous conidiomata, but *Paralloneottiosporina* differs in absent of microconidia, conidia without mucoid appendages, bigger conidia, fewer layers of conidiomatal wall. The macroconidia of *Alloneottiosporina* species are usually accompanied with mucoid appendages at both ends, and microconidia are produced near the ostiolar channel. Moreover, colonies are whitish to bright orange-pink on PDA in *Paralloneottiosporina*, but olivaceous-black in *Alloneottiosporina* [43]. Based on morphological characteristics and molecular phylogeny, the new genus is introduced in Phaeosphaeriaceae.

**Figure 10:**
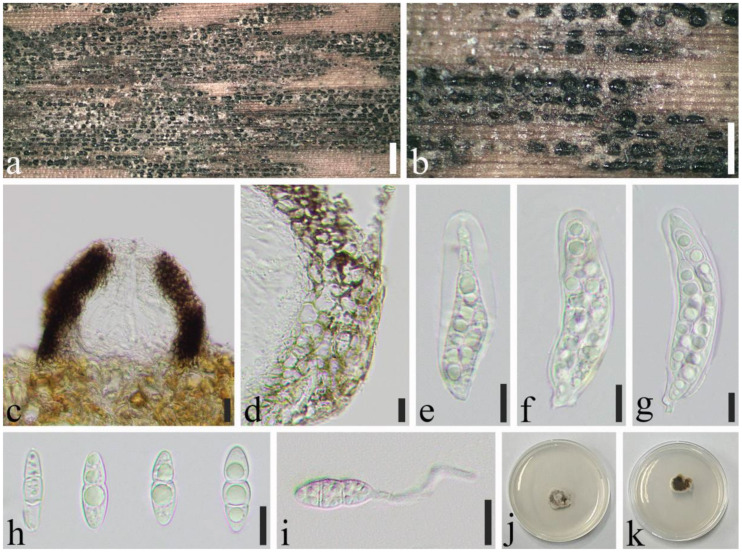
*Paralloneottiosporina sichuanensis* (SICAU 22-0074, holotype). (**a**,**b**) Ascostromata developing on the host. (**c**) Vertical sections of ascostromata. (**d**) Peridium. (**e**–**g**) Asci. (**h**) Ascospores. (**i**) Germinating ascospore. (**j**,**k**) Cultures on PDA. Scale bars: (**a**) = 1 mm, (**b**) = 500 µm, (**c**,**d**) = 20 µm, (**e**–**i**) = 10 µm.

**Figure 11:**
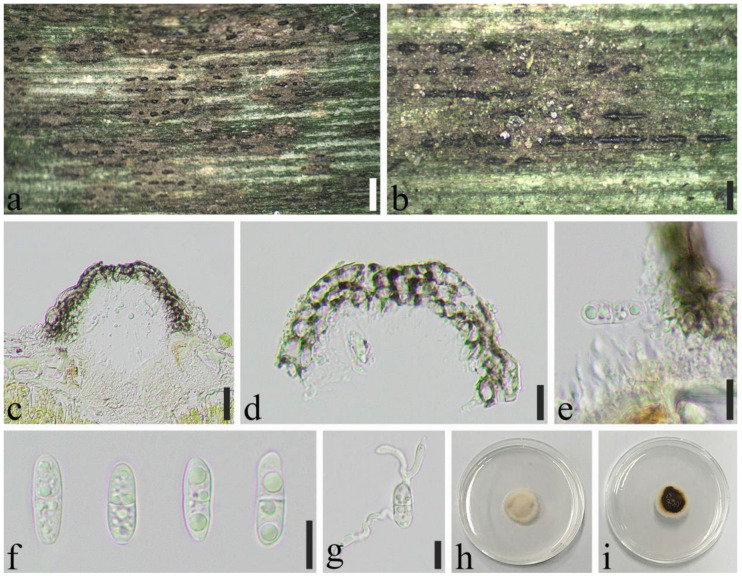
*Paralloneottiosporina sichuanensis* (SICAU 22-0075, paratype). (**a**,**b**) Conidiomata on the host. (**c**) Vertical sections of conidiomata. (**d**) Peridium. (**e**) Conidiogenous cells and developing conidia. (**f**) Conidia. (**g**) Germinating conidium. (**h**,**i**) Cultures on PDA. Scale bars: (**a**) = 500 µm, (**b**) = 200 µm, (**c**) = 20 µm, (**d**–**g**) = 10 µm.

*Paralloneottiosporina sichuanensis* Q. Zeng, Y.C. Lv & C.L. Yang, sp. nov. (Figure 10 and Figure 11).

Index Fungorum: IF559627.

Etymology: In reference to Sichuan Province where the specimens were collected.

Holotype: SICAU 22-0074.

Associated with leaf blight on living to nearly dead leaves of *Phyllostachys violascens* (Poaceae). *Sexual morph*: *Ascomata* 106–343 × 39–196 × 55–112 μm ($\bar{x}$ = 168 × 111 × 89 μm, *n* = 30), separate, gregarious to confluent, globose to subglobose, dark brown to black, superficial, unilocular, glabrous. *Ostiole* single, circular, centrally located. *Peridium* 17–38 μm wide ($\bar{x}$ = 29 μm, *n* = 30), composed of 7–12 layers, with brown cells of *textura angularis*. *Hamathecium* hyaline, dense, cellular, with distinct septa. *Asci* 49–97 × 8.5–19 μm ($\bar{x}$ = 71 × 13 μm, *n* = 30), 8-spored, bitunicate, cylindrical, curved, with a short pedicel. *Ascospores* 15–21 × 5.0–7.5 μm ($\bar{x}$ = 18 × 6.0 μm, *n* = 50), overlapping biseriate, straight, hyaline, fusiform, 1–2 septate, constricted at the septum, smooth-walled, with narrowly rounded ends. *Asexual morph: Conidiomata* 90–191 × 61–132 × 81–123 μm ($\bar{x}$ = 132 × 102 × 105 μm, *n* = 30), globose to long ellipsoid, coriaceous, semi-immersed, black, unilocular, gregarious, glabrous. *Conidiomatal wall* 7.5–21 μm wide ($\bar{x}$ = 13 μm), comprising 3–6 layers, brown cells of *textura angularis*. *Conidiophores* reduced to conidiogenous cells. *Conidiogenous cell* 3.0–6.5 × 2.5–5.0 μm ($\bar{x}$ = 5.0 × 3.5 μm, *n* = 20), hyaline, ampulliform to subcylindrical, smooth. *Conidia* 11–20 × 4.0–6.5 μm ($\bar{x}$ = 17 ×5.0 μm, *n* = 50), ellipsoid to ovoid, 1–septate, slightly constricted at the septum, smooth-walled, hyaline, with a rounded apex and a truncated base.

Material examined: China, Sichuan Province, Ya’an City, Yucheng District (29°56′49.54″ N, 102°56′46.03″ E, Alt. 807 m), on living to nearly dead leaves of *Phyllostachys violascens*, 13 May 2020, Qian Zeng, ZQ202005002 (SICAU 22-0074, holotype), ex-type living culture, SICAUCC 22-0074; CHINA, Sichuan Province, Qionglai City, Linjiang Town (30°19′4.42″ N, 103°17′23.06″ E, Alt. 518 m), on living leaves of *Ph. violascens*, 8 November 2020, Qian Zeng, ZQ202011012 (SICAU 22-0075, paratype), living culture, SICAUCC 22-0075.

Culture characteristics: Ascospores germinate in sterilized water within 24 h at 25 °C. Colonies grow slow on PDA, reaching approximately 2.5 cm in 30 days at 25 °C, circular, white aerial mycelium, whitish to bright orange-pink on the surface, and brown on the back.

Pleosporales Luttr. ex M.E. Barr, Prodromus to class Loculoascomycetes: 67 (1987).

Bambusicolaceae D.Q. Dai & K.D. Hyde, Fungal Diversity. 63 (1): 49 (2013).

**Figure 12:**
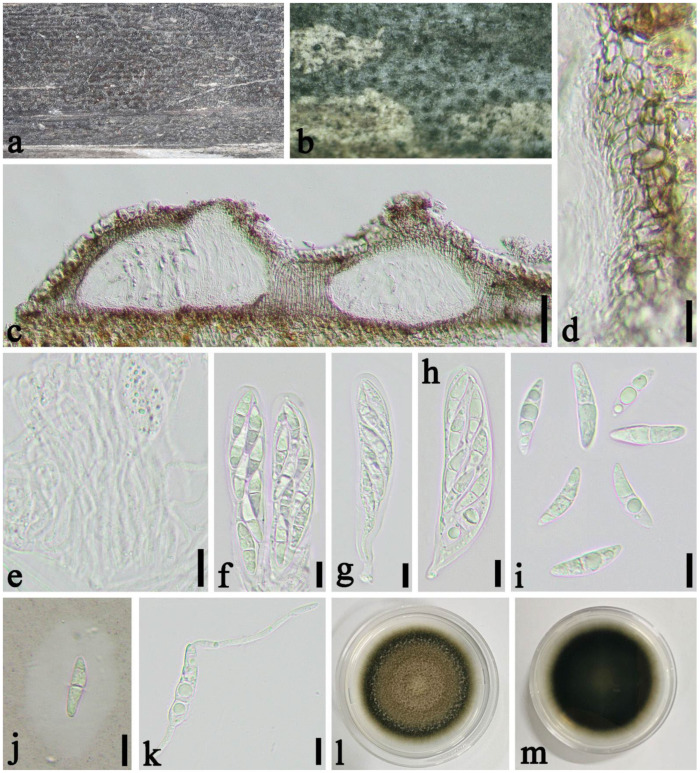
*Seriascoma yunnanense* (SICAU 22-0059). (**a**,**b**) Ascostromata developing on the host. (**c**) Vertical sections of ascostromata. (**d**) Peridium. (**e**) Pseudoparaphyses. (**f**–**h**) Asci. (**i**,**j**) Ascospores. (**k**) Germinating ascospore. (**l**,**m**) Cultures on PDA. Scale bars: (**c**) = 50 µm, (**d**–**k**) = 10 µm.

*Seriascoma yunnanense* Rathnayaka & K.D. Hyde, Asian Journal of Mycology 2(1): 250 (2019) (Figure 12).

Saprobic on dead culm of *Phyllostachys edulis* (Carriere) J. Houzeau. *Sexual morph*: *Ascostromata* 110–200 × 120–150 × 120–140 μm ($\bar{x}$ = 160 × 140 × 130 μm, *n* = 20), solitary to gregarious, immersed, globose to subglobose, coriaceous, dark brown to black. *Peridium* 12–26 μm wide ($\bar{x}$ = 4.0 μm, *n* = 20), composed of 4–9 layers of brown to hyaline cells of *textura angularis*. *Hamathecium* 1.5–2.0 μm wide, composed of dense, branched, long, septate. *Asci* 52–80 × 12–16 μm, ($\bar{x}$ = 60 × 14 μm, *n* = 50), 8-spored, bitunicate, broadly cylindrical, with a short pedicel, straight or slightly curved, with an apical chamber. *Ascospores* 20–30 × 6.0–7.5μm ($\bar{x}$ = 23 × 7.0 μm, *n* = 50), 2–seriate, 1–septate, slightly constricted at the septum, fusiform, narrowly acute at both ends, straight to curved, hyaline, smooth-walled, surrounded by a gelatinous sheath. *Asexual morph*: Undetermined.

Material examined: China, Sichuan Province, Chengdu City, Jin’niu District (30°45′57″ N, 104°7′34″ E, Alt. 539 m), on dead culm of *Phyllostachys edulis*, 8 April 2021, Yicong Lv, LYC202104043 (SICAU 22-0059), living culture SICAUCC 22-0059.

Culture characteristics: Ascospores germinate in sterile water within 12 h at 25 °C. Colonies grow slowly on PDA, and reach 6 cm after 30 days at 25 °C, circular, brown to dark brown.

Notes: On the morphology, our observations were identical to the descriptions of *Seriascoma yunnanense* provided by Rathnayaka et al. [44]. Nucleotide comparisons of SSU, LSU, *tef1-α* and *rpb2* (SICAUCC 22-0059) showed high homology with the sequences of *S. yunnanense* (MFLU 19-0690), similarities are 98.37% (847/861, 0 gaps), 100% (841/841, 0 gaps), 96.59% (396/410, 0 gaps), 99.65% (855/858, 0 gaps), respectively. We report our collection as *S*. *yunnanense*.

## 4. Discussion

In this study, we confirmed seven species of saprophyte or parasitism from leaves and culms of *Phyllostachys*, corresponding to four genera. Microfungi are abundant on culms and leaves of bamboo as pointed out by Dai et al. [45]. Ascomycetes are the most abundant species on bamboo, with about 1150 taxa having been recorded [45]. Furthermore, the number of saprophytic fungi is more than that of pathogenic fungi [16,36].

The genus *Apiospora* Sacc. was recognized and described by Saccardo considering *Ap. montagnei* designated as the type species [46]. *Apiospora* has been widely accepted as a synonym for *Arthrinium* after Ellis [47]. Crous and Groenewald combined *Apiospora* species to be sexual morphs of *Arthrinium* species and synonymized under *Arthrinium* [40]. However, Pintos and Alvarado found that the morphological and ecological differences between *Apiospora* and *Arthrinium* are sufficient to support the taxonomic separation of the two genera. As a result, fifty-five species of *Arthrinium* were combined to *Apiospora* [48]. In this study, given the phylogenetic analysis with species of *Apiospora* and *Arthrinium*, in which 10 species of *Arthrinium* (*Ar.* *agari, Ar. arctoscopi, Ar. fermenti, Ar. koreanum, Ar. mori, Ar. phaeospermum, Ar. pusillispermum, Ar. sargassi, Ar. taeanense, Ar. marinum*) are clustered in a well-supported clade within *Apiospora*, future studies are needed to better understand the combination of previous *Arthrinium* species with *Apiospora. Apiospora* species have a worldwide distribution and can be found on various hosts. Most species occurred on the plants in Poaceae, although some were known from Amaranthaceae, Juncaceae, Euphorbiaceae, Cyperaceae, Restionaceae, Fagaeaeand, even seaweeds [48,49]. To date, more than 25 species have been found on bamboo, most species were saprobic on dead bamboo culms, and a few species have been reported as pathogens. For example, *Ap. arundinis* causes brown culm streak of *Phyllostachys praecox,* and *Ap. kogelbergensis* causes blight disease of *Bambusa intermedia* [16,41,50,51]. *Apiospora. hydei*, *Ap. neosubglobosa*, and *Ap. jiangxiensis* were saprophytic on unidentified bamboo culms and leaves [41,52]. *Apiospora yunnansis* has been reported on bamboo culms of *Phyllostachys nigra* and *P. heteroclada*, which can cause bamboo blight disease of *P. heteroclada* [53,54]. In this study, four known species, *Apiospora* *hydei*, *Ap. neosubglobosa*, *Ap. jiangxiensis,* *and Ap. yunnansis,* were newly recorded on *Phyllostachys* *nigra*, *P*. *heteroclada*, *P*. *bissetii,* and *P*. *aurea* respectively.

At present, *Bifusisporella* only comprises the ex-type species *B. sorghi*. In this study, we provide taxonomic details for another new species, *B. sichuanensis*, that was collected from living leaves of *Phyllostachys edulis*. *B. sorghi* was isolated as an endophyte from healthy sorghum leaves in Brazil by Silva et al. [42]. However, *B. sichuanensis* is pathogenic, causing tar spot on bamboo leaves. In addition, the sexual stage in this genus is supplemented.

Phaeosphaeriaceae is one of the most important and species-rich families in Pleosporales with diverse lifestyles [55,56], and may be found on herbaceous stems or monocotyledonous culms, branches, leaves, flowers, and woody substrates [57,58]. Currently, more than 70 genera are accommodated in Phaeosphaeriaceae [59]. Most genera in this family were introduced as monotypic genera, such as *Acericola*, *Banksiophoma*, *Bhagirathimyces, Bhatiellae, Brunneomurispora, Camarosporioides, Elongaticollum, Equiseticola, Hydeopsis, Jeremyomyces, Mauginiella, Melnikia, Neoophiobolus, Neosphaerellopsis, Neostagonosporella, Ophiobolopsis,* and *Parastagonosporella*, among others. Due to these genera being represented by a single species, resulting in few samples that could be used for taxon, the phylogenetic relationships with the related genera are sometimes not well-resolved. Based on morphological characteristics and multigene phylogeny, a novel genus, *Paralloneottiosporina,* is introduced to accommodate *Pa. sichuanensis* sp. nov. According to the field investigation, *Pa. sichuanensis* can cause leaf blight that eventually leads to leaf necrosis and plant decline in severe cases. Besides *Ph*. *violascens*, leaf blight caused by *Pa. sichuanensis* has also been observed on *P. heterocycla* and *P. tianmuensis*. This indicates that *Pa. sichuanensis* may be a common parasitic fungus on bamboos.

As only three species are accommodated within *Seriascoma*, more research is also needed for better understanding this genus [60]. *Seriascoma* is presently known as saprobic on decaying wood and dead bamboo in the terrestrial or freshwater habitats distributed in China and Thailand [16,44,61,62]. *Seriascoma. yunnanense* is found on dead branches of bamboo in Yunnan. In this study, *S. yunnanense* was saprophytic on *Phyllostachys* *edulis*.

The previous studies have revealed a high fungal diversity associated with bamboo *Phyllostachys*. In recent years, 10 species belonging to seven genera have been described from bamboo of *Phyllostachys*, including two new genera, *Neostagonosporella* and *Parakarstenia,* established by Yang et al. on *P. heteroclada* in Sichuan Province [54,58,63,64,65,66,67,68,69]. However, the knowledge about bambusicolous fungi is incomplete and mainly remains at cataloguing stage [14]. The previous studies of identification were mostly based on morphological characteristics, and lacked molecular data. Moreover, their hosts were poorly documented or unknown [70], and specimens were absent for further re-examination. Therefore, these species need to be recollected, epitypified, and sequenced [10], and new species need to be discovered and described.

## Figures and Tables

**Table 1:** Selected genes for polymerase chain reaction of each genus.

Genera	Sequences Dataset
*Apiospora*	ITS, LSU, *tub2*, *tef1-α*
*Bifusisporella*	ITS, LSU, *tef1-α*, *rpb1*
*Paralloneottiosporina*	ITS, LSU, SSU, *tef1-α*
*Seriascom*	ITS, LSU, SSU, *tef1-α*, *rpb2*

## Data Availability

The datasets presented in this study can be found in the NCBI GenBank (https://www.ncbi.nlm.nih.gov/), Index Fungorum (http://www.indexfungorum.org/Names/Names.asp) (all accessed on 8 May 2022).

## Appendix A. Molecular Data Used in This Study and GenBank Accession Numbers

**Table A1:** Isolates and GenBank accession numbers of sequences used in this study.

Species	Strains	GenBank Accession Numbers							
		ITS	LSU	*tub2*	*tef1-a*	*rpb1*	SSU	*rpb2*	References
*Apiospora acutiapica*	KUMCC 20-0209	MT946342	MT946338	–	–	–	–	–	[71]
*Apiospora acutiapica*	KUMCC 20-0210 ^T^	MT946343	MT946339		–	–	–	–	[71]
*Apiospora aquaticum*	MFLU 18-1628 ^T^	MK828608	MK835806	–	–	–	–	–	[55]
*Apiospora arundinis*	CBS 114316	KF144884	KF144928	KF144974	KF145016	–	–	–	[40]
*Apiospora arundinis*	CBS 450.92	AB220259	–	AB220306	–	–	–	–	[71]
*Apiospora arundinis*	AP11118A	MK014868	MK014835	MK017974	MK017945	–	–	–	[72]
*Apiospora aureum*	CBS 244.83 ^T^	AB220251	KF144935	KF144981	KF145023	–	–	–	NCBI
*Apiospora balearica*	CBS 145129 ^T^	MK014869	MK014836	MK017975	MK017946	–	–	–	[72]
*Apiospora bambusicola*	MFLUCC 20-0144 ^T^	MW173030	MW173087	–	MW183262	–	–	–	[73]
*Apiospora biserialis*	CGMCC 3.20135 ^T^	MW481708	–	MW522955	MW522938	–	–	–	[52]
*Apiospora camelliae-sinensis*	LC5007 ^T^	KY494704	KY494780	KY705173	KY705103	–	–	–	[36]
*Apiospora camelliae-sinensis*	LC8181	KY494761	KY494837	KY705229	KY705157	–	–	–	[36]
*Apiospora chiangraiense*	MFLU:21-0046	MZ542520	MZ542524	MZ546409	–	–	–	–	[49]
*Apiospora chromolaenae*	MFLUCC 17-1505 ^T^	MT214342	MT214436	–	–	–	–	–	[74]
*Apiospora cyclobalanopsidis*	CGMCC 3.20136 ^T^	MW481713	–	MW522962	MW522945	–	–	–	[52]
*Apiospora descalsii*	CBS 145130 ^T^	MK014870	MK014837	MK017976	MK017947	–	–	–	[72]
*Apiospora dichotomanthi*	CGMCC 3.18332 ^T^	KY494697	KY494832	KY705167	KY705096	–	–	–	[36]
*Apiospora dichotomanthi*	LC8175	KY494755	KY494831	KY705223	KY705151	–	–	–	[36]
*Apiospora esporlensis*	CBS 145136 ^T^	MK014878	MK014845	MK017983	MK017954	–	–	–	[72]
*Apiospora euphorbiae*	IMI 285638b	AB220241	–	AB220288	–	–	–	–	[71]
*Apiospora gaoyouensis*	CFCC 52301	MH197124	–	MH236789	MH236793	–	–	–	[53]
*Apiospora gaoyouensis*	CFCC 52302	MH197125	–	MH236790	MH236794	–	–	–	[53]
*Apiospora garethjonesii*	JHB004	KY356086	KY356091	–	–	–	–	–	[41]
*Apiospora garethjonesii*	HKAS 96289 ^T^	NR_154736	NG_057131	–	–	–	–	–	[41]
*Apiospora gelatinosa*	HKAS:111962	–	–	MW5229	MW522941	–	–	–	[52]
*Apiospora guizhouensis*	LC5318	KY494708	KY494784	KY705177	KY705107	–	–	–	[36]
*Apiospora guizhouensis*	CGMCC 3.18334 ^T^ = LC5322	KY494709	KY494785	KY705178	KY705108	–	–	–	[36]
*Apiospora hispanica*	IMI 326877	AB220242	AB220336	AB220289	–	–	–	–	[71]
*Apiospora hydei*	CBS 114990 ^T^	KF144890	KF144936	KF144982	KF145024	–	–	–	[40]
*Apiospora hydei*	KUMCC 16-0204	KY356087	KY356092	–	–	–	–	–	[41]
*Apiospora hydei*	**SICAUCC 22-0032**	**ON183998**	**ON185553**	**ON221313**	**ON221312**	–	–	–	This study
*Apiospora hyphopodii*	MFLUCC 15-003 ^T^	KR069110	KR069111	–	–	–	–	–	[75]
*Apiospora hyphopodii*	KUMCC 16-0201	KY356088	KY356093	–	–	–	–	–	[41]
*Apiospora hysterina*	AP15318	MK014873	MK014840	MK017979	MK017950	–	–	–	[72]
*Apiospora hysterina*	ICPM6889	MK014874	MK014841	MK017980	MK017951	–	–	–	[72]
*Apiospora hysterina*	AP29717	MK014875	MK014842	MK017981	MK017952	–	–	–	[72]
*Apiospora hysterina*	AP2410173	MK014876	MK014843	–	–	–	–	–	[72]
*Apiospora hysterina*	AP12118	MK014877	MK014844	MK017982	MK017953	–	–	–	[72]
*Apiospora iberica*	CBS 145137 ^T^	MK014879	MK014846	MK017984	MK017955	–	–	–	[72]
*Apiospora intestini*	CBS 135835 ^T^	KR011352	MH877577	KR011350	KR011351	–	–	–	[76]
*Apiospora italica*	CBS 145138 ^T^	MK014880	MK014847	MK017985	MK017956	–	–	–	[72]
*Apiospora italica*	AP221017 ^T^	MK014881	MK014848	MK017986	MK017957	–	–	–	[72]
*Apiospora jatrophae*	CBS 134262 ^T^	NR_154675	–	–	–	–	–	–	[77]
*Apiospora jatrophae*	MMI 00051 = CBS:134262	JQ246355	–	–	–	–	–	–	[77]
*Apiospora jiangxiensis*	CGMCC 3.18381 ^T^	KY494693	–	KY705163	KY705092	–	–	–	[36]
*Apiospora jiangxiensis*	LC4578	KY494694	KY494770	KY705164	KY705093	–	–	–	[36]
***Apiospora jiangxiensis***	**SICAUCC 22-0070**	**ON227094**	**ON227098**	**ON244432**	**ON244431**	–	–	–	This study
*Apiospora kogelbergensis*	CBS 113332	KF144891	KF144937	KF144983	KF145025	–	–	–	[40]
*Apiospora kogelbergensis*	CBS 113333 ^T^	KF144892	KF144938	KF144984	KF145026	–	–	–	[40]
*Apiospora kogelbergensis*	CBS 113335	KF144893	KF144939	KF144985	KF145027	–	–	–	[40]
*Apiospora kogelbergensis*	CBS 117206	KF144895	KF144941	KF144987	KF145029	–	–	–	[40]
*Apiospora locuta-pollinis*	LC11683	MF939595	–	MF939622	MF939616	–	–	–	[78]
*Apiospora longistroma*	MFLUCC 11-0479	KU940142	KU863130	–	–	–	–	–	[16]
*Apiospora longistroma*	MFLUCC 11-0481	KU940141	KU863129	–	–	–	–	–	[16]
*Apiospora longistroma*	MFLU 15-1184 ^T^	NR_154716	–	–	–	–	–	–	[16]
*Apiospora malaysiana*	CBS 102053 ^T^	KF144896	KF144942	KF144988	KF145030	–	–	–	[40]
*Apiospora marii*	CBS 497.90 ^T^	AB220252	KF144947	KF144993	KF145035	–	–	–	[40]
*Apiospora mediterranea*	IMI 326875	AB220243	–	AB220290	–	–	–	–	[71]
*Apiospora minutispora*	17E-042	LC517882	–	LC518888	LC518889	–	–	–	[79]
*Apiospora montagnei*	LSU0093	MT000394	MT000490	–	–	–	–	–	[80]
*Apiospora mytilomorpha*	DAOM 214595	KY494685	–	–	–	–	–	–	[36]
*Apiospora neobambusae*	CGMCC 3.18335 ^T^	KY494718	KY494794	KY705186	KY806204	–	–	–	[36]
*Apiospora neobambusae*	KUMCC 20-0207	MT946346	MT946340	–	–	–	–	–	[71]
*Apiospora neobambusae*	LC7107	KY494719	KY494795	KY705187	KY705117	–	–	–	[36]
*Apiospora neochinensis*	CFCC 53037	MK819292	–	MK818548	MK818546	–	–	–	[81]
*Apiospora neochinensis*	CFCC 53036 ^T^	MK819291	–	MK818547	MK818545	–	–	–	[81]
*Apiospora neogarethjonesii*	HKAS 96354 ^T^	MK070897	MK070898	–	–	–	–	–	[82]
*Apiospora neosubglobosa*	JHB006	KY356089	KY356094	–	–	–	–	–	[41]
*Apiospora neosubglobosa*	JHB007 ^T^	KY356090	KY356095	–	–	–	–	–	[41]
***Apiospora neosubglobosa***	**SICAUCC 22-0071**	**ON227095**	**ON227099**	**ON244430**	**ON244429**	–	–	–	This study
*Apiospora obovata*	CGMCC 3.18331 ^T^	KY494696	KY494834	KY705166	KY705095	–	–	–	[41]
*Apiospora obovata*	LC8177	KY494757	KY494833	KY705225	KY705153	–	–	–	[41]
*Apiospora ovata*	CBS 115042 ^T^	KF144903	KF144950	KF144995	KF145037	–	–	–	[40]
*Arthrinium paraphaeospermum*	NCYU 19-0341	MW114315	MW293936	–	MW288020	–	–	–	NCBI
*Apiospora paraphaeosperma*	MFLUCC 13-0644 ^T^	KX822128	KX822124	–	–	–	–	–	[71]
*Apiospora phragmitis*	CPC 18900 ^T^	KF144909	–	KF145001	KF145043	–	–	–	[40]
*Apiospora phragmitis*	AP3218	MK014891	MK014858	MK017996	MK017967	–	–	–	[72]
*Apiospora phragmitis*	AP2410172A	MK014890	MK014857	MK017995	MK017966	–	–	–	[72]
*Apiospora phyllostachydis*	MFLUCC 18-1101	–	–	MK291949	–	–	–	–	[65]
*Apiospora piptatheri*	CBS 145149 ^T^	MK014893	MK014860	–	MK017969	–	–	–	[72]
*Apiospora pseudoparenchymatica*	CGMCC 3.18336 ^T^	KY494743	KY494819	KY705211	KY705139	–	–	–	[36]
*Apiospora pseudoparenchymatica*	LC8173	KY494753	KY494829	KY705221	KY705149	–	–	–	[36]
*Apiospora pseudorasikravindrae*	KUMCC 20-0208 ^T^	MT946344	–	–	–	–	–	–	[71]
*Apiospora pseudorasikravindrae*	KUMCC 20-0211	MT946345	–	–	–	–	–	–	[71]
*Apiospora pseudosinensis*	CBS 135459 ^T^	KF144910	KF144957	–	KF145044	–	–	–	[40]
*Apiospora pseudospegazzinii*	CBS 102052 ^T^	KF144911	KF144958	KF145002	KF145045	–	–	–	[40]
*Apiospora pterosperma*	CBS 123185	KF144912	KF144959	KF145003		–	–	–	[40]
*Apiospora pterosperma*	CBS 134000 ^T^	KF144913	KF144960	KF145004	KF145046	–	–	–	[40]
*Apiospora qinlingensis*	CFCC 52303 ^T^	MH197120	–	MH236791	MH236795	–	–	–	[53]
*Apiospora qinlingensis*	CFCC 52304	MH197121	–	MH236792	MH236796	–	–	–	[53]
*Apiospora rasikravindrae*	NFCCI 2144 ^T^	KF144914	–	–	–	–	–	–	[83]
*Apiospora rasikravindrae*	MFLUCC 11-0616	KU940144	KU863132	–	–	–	–	–	[16]
*Apiospora rasikravindrae*	LC5449	KY494713	KY494789	KY705182	KY705112	–	–	–	[36]
*Apiospora rasikravindrae*	LC7115	KY494721	KY494797	KY705189	KY705118	–	–	–	[36]
*Apiospora rasikravindrae*	KUC21351	MH498540		MH498498	MN868932	–	–	–	[84]
*Apiospora rasikravindrae*	KUC21327	MH498541		MH498499	MH544670	–	–	–	[84]
*Apiospora sacchari*	CBS 212.30	KF144916	KF144962	KF145005	KF145047	–	–	–	[40]
*Apiospora sacchari*	CBS 301.49	KF144917	KF144963	KF145006	KF145048	–	–	–	[40]
*Apiospora saccharicola*	CBS 191.73	KF144920	KF144966	KF145009	KF145051	–	–	–	[40]
*Apiospora saccharicola*	CBS 463.83	KF144921	KF144968	KF145010	KF145052	–	–	–	[40]
*Apiospora sasae*	CBS 146808 ^T^	MW883402	MW883797	MW890120	MW890104	–	–	–	[85]
*Apiospora septatum*	CGMCC 3.20134 ^T^	MW481711	–	MW522960	MW522943	–	–	–	[52]
*Apiospora serenensis*	IMI 326869 ^T^	AB220250	–	AB220297	–	–	–	–	[71]
*Apiospora serenensis*	ATCC 76309	AB220240	–	AB220287	–	–	–	–	[71]
*Apiospora setariae*	CFCC 54041	MT492004	–	MT497466	MW118456	–	–	–	[86]
*Apiospora setostroma*	KUMCC 19-0217	MN528012	MN528011	–	MN527357	–	–	–	[87]
*Apiospora sinensis*	UNKNOW-1 = HKUCC 3143	–	AY083831	–	–	–	–	–	NCBI
*Apiospora sinensis*	UNKNOW-2	–	DQ810215	–	–	–	–	–	NCBI
*Apiospora sorghi*	URM<BRA>:9300	MK371706	–	–	–	–	–	–	NCBI
*Apiospora stipae*	CBS 146804	MW883403	MW883798	MW890121	MW890105	–	–	–	[85]
*Apiospora subglobosa*	MFLUCC 11-0397 ^T^	KR069112	KR069113	–	–	–	–	–	[75]
*Apiospora subrosea*	LC7291	KY494751	KY494827	KY705219	KY705147	–	–	–	[36]
*Apiospora subrosea*	CGMCC3.18337 ^T^	KY494752	KY494828	KY705220	KY705148	–	–	–	[36]
*Apiospora thailandica*	MFLUCC 15-0199	KU940146	KU863134	–	–	–	–	–	[16]
*Apiospora thailandica*	MFLUCC 15-0202 ^T^	KU940145	KU863133	–	–	–	–	–	[16]
*Apiospora thailandica*	LC5630	KY494714	KY494790	KY806200	KY705113	–	–	–	[36]
*Apiospora tintinnabula*	7019-96 (ICMP)	–	DQ810216	–	–	–	–	–	[71]
*Apiospora vietnamensis*	IMI 99670	KX986096	KX986111	KY019466	–	–	–	–	[88]
*Apiospora xenocordella*	CBS 478.86 ^T^	KF144925	KY494763	–	–	–	–	–	[40]
*Apiospora xenocordella*	CBS 595.66	KF144926	KF144971	KF145013	KF145055	–	–	–	[40]
*Apiospora yunnana*	MFLUCC 15-0002 ^T^	KU940147	KU863135	–	–	–	–	–	[16]
***Apiospora yunnana***	**SICAUCC 22-0072**	**ON227096**	**ON227100**	**ON244426**	**ON244425**	–	–	–	This study
*Arthrinium agari*	KUC21364	MH498516	–	MH498474	MN868917	–	–	–	[84]
*Arthrinium arctoscopi*	KUC21347	MH498525	–	MH498483	MN868922	–	–	–	[84]
*Arthrinium fermenti*	KUC21289	MF615226	–	MF615231	MH544667	–	–	–	[84]
*Arthrinium koreanum*	KUC21350	MH498521	–	MH498479	MN868929	–	–	–	[84]
*Arthrinium marinum*	KUC21328	MH498538		MH498496	MH544669				[84]
*Arthrinium marinum*	KUC21356	MH498534	–	MH498492	MN868926	–	–	–	[84]
*Arthrinium marinum*	KUC21355	MH498535		MH498493	MN868925				[84]
*Arthrinium marinum*	KUC21354	MH498536		MH498494	MN868924				[84]
*Arthrinium mori*	MFLU 18-2514	MW114313	MW114393	–	–	–	–	–	[89]
*Arthrinium mori*	NCYU 19-0364	MW114314	MW114394	–	–	–	–	–	[89]
*Arthrinium phaeospermum*	CBS 114315	KF144905	KF144952	KF144997	KF145039	–	–	–	[40]
*Arthrinium phaeospermum*	CBS 114317	KF144906	KF144953	KF144998	KF145040	–	–	–	[40]
*Arthrinium phaeospermum*	CBS 114318	KF144907	KF144954	KF144999	KF145041	–	–	–	[40]
*Arthrinium pusillispermum*	KUC21357	MH498532	–	MH498490	MN868931	–	–	–	[84]
*Arthrinium sargassi*	KUC21232	KT207750	–	KT207648	MH544676	–	–	–	[84]
*Arthrinium taeanense*	KUC21322	MH498515	–	MH498473	MH544662	–	–	–	[84]
*Pestalotiopsis chamaeropis*	CBS 237.38	MH855954	MH867450	KM199392	KM199474	–	–	–	[76]
*Pestalotiopsis colombiensis*	CBS 118553 ^T^	KM199307	KM116222	KM199421	KM199488	–	–	–	[90]
*Bambusicularia brunnea*	CBS 133599 ^T^	KM484830	KM484948	–	–	KM485043	–	–	[91]
*Bambusicularia brunnea*	CBS 133600	AB274436	KM484949	–	–	KM485044	–	–	[91,92]
*Barretomyces calatheae*	CBS 129274 = CPC 18464	KM484831	KM484950	–	–	KM485045	–	–	[76]
***Bifusisporella sichuanensis***	**SICAUCC 22-0073** **^T^**	**ON227097**	**ON227101**	**–**	**ON244427**	ON244428	–	–	This study
*Bifusisporella sorghi*	URM 7442 ^T^	MK060155	MK060153	–	MK060157	MK060159	–	–	[42]
*Bifusisporella sorghi*	URM 7864	MK060156	MK060154	–	MK060158	MK060160	–	–	[42]
*Buergenerula spartinae*	ATCC 22848	JX134666	DQ341492	–	JX134692	JX134720	–	–	[93]
*Bussabanomyces longisporus*	CBS 125232 ^T^	KM484832	KM484951	–	KM009202	KM485046	–	–	[94]
*Falciphora oryzae*	CBS 125863 ^T^	EU636699	KJ026705	–	JN857963	KJ026706	–	–	[95]
*Falciphoriella solaniterrestris*	CBS 117.83 ^T^	KM484842	KM484959	–	–	KM485058	–	–	[91]
*Gaeumannomycella caricicola*	CBS:145041	MK442584	MK442526	–	–	–	–	–	[96]
*Gaeumannomycella caricis*	CBS 388.81 ^T^	KM484843	KM484960	–	KX306674	–	–	–	[91]
*Gaeumannomyces australiensis*	CPC 26058 ^T^	KX306480	KX306550	–	KX306683	KX306619	–	–	[97]
*Gaeumannomyces avenae*	CBS 187.65	JX134668	JX134680	–	–	JX134722	–	–	[93]
*Gaeumannomyces avenae*	CBS 870.73 = DAR 20999	KM484833	DQ341495	–	–	KM485048	–	–	[91]
*Gaeumannomyces californicus*	CPC 26044 ^T^	KX306490	KX306560	–	KX306691	KX306625	–	–	[97]
*Gaeumannomyces ellisiorum*	CBS 387.81 ^T^	KM484835	KM484952	–	KX306692	KM485051	–	–	[91]
*Gaeumannomyces floridanus*	CPC 26037 ^T^	KX306491	KX306561	–	KX306693	KX306626	–	–	[97]
*Gaeumannomyces fusiformis*	CPC 26068 ^T^	KX306492	KX306562	–	KX306694	KX306627	–	–	[97]
*Gaeumannomyces glycinicola*	CPC 26266	KX306494	KX306564	–	KX306696	KX306629	–	–	[97]
*Gaeumannomyces glycinicola*	CPC 26057	KX306493	KX306563	–	KX306695	KX306628	–	–	[97]
*Gaeumannomyces graminicola*	CBS 352.93 ^T^	KM484834	DQ341496	–	KX306697	KM485050	–	–	[91]
*Gaeumannomyces graminis*	CPC 26045	KX306505	KX306575	–	KX306708	KX306640	–	–	[97]
*Gaeumannomyces graminis var. graminis*	M33	JF710374	JF414896	–	JF710411	JF710442	–	–	[98]
*Gaeumannomyces graminis var. graminis*	M54	JF414848	JF414898	–	JF710419	JF710444	–	–	[98]
*Gaeumannomyces hyphopodioides*	CBS 350.77 ^T^	KX306506	KX306576	–	–	–	–	–	[97]
*Gaeumannomyces hyphopodioides*	CBS 541.86	KX306507	KX306577	–	KX306709	–	–	–	[97]
*Gaeumannomyces oryzicola*	CPC 26063 ^T^	KX306516	KX306586	–	KX306717	KX306646	–	–	[97]
*Gaeumannomyces oryzinus*	CPC 26030 ^T^	KX306517	KX306587	–	KX306718	KX306647	–	–	[97]
*Gaeumannomyces radicicola*	CBS 296.53 ^T^	KM009170	KM009158	–	KM009206	KM009194	–	–	[94]
*Gaeumannomyces setariicola*	CPC 26059	KX306524	KX306594	–	KX306725	KX306654	–	–	[97]
*Gaeumannomyces tritici*	CBS 273.36	KX306525	KX306595	–	KX306729	KX306655	–	–	[97]
*Gaeumannomyces walkeri*	CPC 26028 ^T^	KX306543	KX306613	–	KX306746	KX306670	–	–	[97]
*Gaeumannomyces wongoonoo*	BRIP:60376	KP162137	KP162146	–	–	–	–	–	[99]
*Kohlmeyeriopsis medullaris*	CBS 117849 ^T^ = JK5528S	KM484852	KM484968	–	–	KM485068	–	–	[91]
*Macgarvieomyces borealis*	CBS 461.65 ^T^	MH858669	DQ341511	–	KM009198	KM485070	–	–	[94]
*Macgarvieomyces juncicola*	CBS 610.82	KM484855	KM484970	–	KM009201	KM485071	–	–	[91]
*Magnaporthiopsis agrostidis*	BRIP 59300 ^T^	KT364753	KT364754	–	KT364756	KT364755	–	–	[100]
*Magnaporthiopsis cynodontis*	RS7-2 = CBS 141700 ^T^	KJ855508	KM401648	–	KP282714	KP268930	–	–	[101]
*Magnaporthiopsis cynodontis*	RS5-5	KJ855506	KM401646	–	KP282712	KP268928	–	–	[101]
*Magnaporthiopsis cynodontis*	RS3-1	KJ855505	KM401645	–	KP282711	KP268927	–	–	[101]
*Magnaporthiopsis incrustans*	M35	JF414843	JF414892	–	JF710412	JF710437	–	–	[98]
*Magnaporthiopsis maydis*	M84	KM009160	KM009148	–	KM009196	KM009184	–	–	[94]
*Magnaporthiopsis maydis*	M85	KM009161	KM009149	–	KM009197	KM009185	–	–	[94]
*Magnaporthiopsis meyeri-festucae*	FF2	MF178146	MF178151	–	MF178167	MF178162	–	–	[102]
*Magnaporthiopsis meyeri-festucae*	SCR11	MF178150	MF178155	–	MF178171	MF178166	–	–	[102]
*Magnaporthiopsis panicorum*	CM2S8 ^T^	KF689643	KF689633	–	KF689623	KF689613	–	–	[103]
*Magnaporthiopsis panicorum*	CM10s2	KF689644	KF689634	–	KF689624	KF689614	–	–	[103]
*Magnaporthiopsis poae*	TAP35	KJ855511	KM401651	–	KP282717	KP268933	–	–	[104]
*Magnaporthiopsis poae*	M1	JF414827	JF414876	–	JF710400	JF710425	–	–	[98]
*Magnaporthiopsis poae*	M12	JF414828	JF414877	–	JF710401	JF710426	–	–	[98]
*Magnaporthiopsis rhizophila*	M22	JF414833	JF414882	–	JF710407	JF710431	–	–	[98]
*Nakataea oryzae*	M21	JF414838	JF414887	–	JF710406	JF710441	–	–	[98]
*Nakataea oryzae*	M69	JX134672	JX134685	–	JX134698	JX134726	–	–	[93]
*Nakataea oryzae*	M71	JX134673	JX134686	–	JX134699	JX134727	–	–	[93]
*Neogaeumannomyces bambusicola*	MFLUCC11-0390 ^T^	KP744449	KP744492	–	–	–	–	–	[105]
*Neopyricularia commelinicola*	CBS 128307 = KACC 44083	FJ850125	KM484984	–	KM009199	KM485086	–	–	[91,106]
*Neopyricularia commelinicola*	CBS 128308 ^T^	FJ850122	KM484985	–	–	KM485087	–	–	[91,106]
*Ophioceras dolichostomum*	CBS 114926 = HKUCC 3936 = KM 8	JX134677	JX134689	–	JX134703	JX134731	–	–	[93]
*Ophioceras leptosporum*	CBS 894.70 ^T^ = ATCC 24161 = HME 2955	JX134678	JX134690	–	JX134704	JX134732	–	–	[83]
*Proxipyricularia zingiberis*	CBS 132355 = MAFF 240221	AB274433	KM484987	–	–	KM485090	–	–	[91]
*Pseudophialophora eragrostis*	CM12m9	KF689648	KF689638	–	KF689628	KF689618	–	–	[103]
*Pseudopyricularia cyperi*	CBS 133595 ^T^ = MAFF 240229	KM484872	KM484990	–	–	AB818013	–	–	[91]
*Pseudopyricularia kyllingae*	CBS 133597 ^T^ = MAFF 240227	KM484876	KM484992	–	KT950880	KM485096	–	–	[91]
*Pyricularia ctenantheicola*	GR0001 = Ct-4 = ATCC 200218	KM484878	KM484994	–	–	KM485098	–	–	[91]
*Pyricularia grisea*	BR0029	KM484880	KM484995	–	–	KM485100	–	–	[91]
*Pyricularia grisea*	CR0024	KM484882	KM484997	–	–	KM485102	–	–	[91]
*Pyricularia oryzae*	CBS 365.52 = MUCL 9451	KM484890	KM485000	–	–	KM485110	–	–	[76]
*Slopeiomyces cylindrosporus*	BAN-145	JF508361	–	–	–	–	–	–	[107]
*Slopeiomyces cylindrosporus*	CG340	AY428776	–	–	–	–	–	–	[108]
*Utrechtiana cibiessia*	CBS 128780 = CPC 18916	JF951153	JF951176	–	–	KM485047	–	–	[76]
*Xenopyricularia zizaniicola*	CBS 132356	KM484946	KM485042	–	KM009203	KM485160	–	–	[91]
*Acericola italica*	MFLUCC 13-0609 ^T^	MF167428	MF167429	–	–	–	MF167430	–	[109]
*Alloneottiosporina thailandica*	MFLUCC 15-0576 ^T^	MT177913	MT177940	–	MT454002	–	MT177968	–	[43]
*Allophaeosphaeria muriformia*	MFLUCC 13-0349 ^T^	KP765680	KP765681	–	–	–	KP765682	–	[105]
*Amarenographium ammophilae*	MFLUCC 16-0296	KU848196	KU848197	–	MG520894	–	KU848198	–	[109]
*Amarenomyces dactylidis*	MFLU 17-0498 ^T^	KY775577	KY775575	–	–	–	–	–	[110]
*Ampelomyces quisqualis*	CBS 129.79 ^T^	–	EU754128	–	–	–	EU754029	–	[111]
*Banksiophoma australiensis*	CBS 142163 ^T^	KY979739	KY979794	–	KY979889	–	–	–	[112]
*Bhagirathimyces himalayensis*	AMH 10127 ^T^ = NFCCI 4580	MK836021	MK836020	–	–	–	MN121697	–	[113]
*Bhatiellae rosae*	MFLUCC 17-0664 ^T^	MG828873	MG828989	–	–	–	MG829101	–	[114]
*Brunneomurispora lonicerae*	KUMCC 18-0157 ^T^	MK356373	MK356346	–	MK359065	–	MK356360	–	[59]
*Camarosporioides phragmitis*	MFLUCC 13-0365 ^T^	KX572340	KX572345	–	KX572354	–	KX572350	–	[115]
*Chaetosphaeronema achilleae*	MFLUCC 16-0476 ^T^	KX765265	KX765266	–	–	–		–	[115]
*Chaetosphaeronema hispidulum*	MFLU:16-1965	MT177915	MT177942	–	–	–	MT177970	–	[43]
*Chaetosphaeronema hispidulum*	MFLU:16-2275	MT177914	MT177941	–	MT454003	–	MT177969	–	[43]
*Chaetosphaeronema hispidulum*	CBS 216.75	KF251148	KF251652	–	KF253108	–		–	[116]
*Dactylidina dactylidis*	MFLUCC 13-0618	KP744432	KP744473	–	–	–	KP753946	–	[105]
*Dactylidina dactylidis*	MFLUCC 14-0966 ^T^	MG828886	MG829002	–	MG829199	–	MG829113	–	[114]
*Dematiopleospora donetzica*	MFLU 15-2199 ^T^	–	MG829005	–	–	–	MG829116	–	[114]
*Dematiopleospora mariae*	MFLUCC 13-0612 ^T^	KJ749654	KJ749653	–	KJ749655	–	KJ749652	–	[117]
*Diederichomyces ficuzzae*	CBS 128019	KP170647	–	–	KP170673	–	–	–	[118]
*Diederichomyces xanthomendozae*	CBS 129666	KP170651	–	–	KP170677	–	–	–	[118]
*Dlhawksworthia clematidicola*	MFLUCC 17-2151 ^T^	MT310619	MT214574	–	MT394633	–	MT226687	–	[119]
*Edenia gomezpompae*	JLCC 34533	KC193601	–	–	–	–	–	–	[120]
*Elongaticollum hedychii*	MFLUCC 18-1638 ^T^	MT321796	MT321810	–	MT328753	–	MT321803	–	[115]
*Elongaticollum hedychii*	NCYUCC 19-0286	MT321797	MT321811	–	MT328754	–	MT321804	–	[115]
*Embarria clematidis*	MFLUCC 14-0652	KT306949	KT306953	–	–	–	KT306956	–	[121]
*Embarria clematidis*	MFLUCC 14-0976	MG828871	MG828987	–	MG829194	–	MG829099	–	[114]
*Equiseticola fusispora*	MFLUCC 14-0522 ^T^	KU987668	KU987669	–	MG520895	–	KU987670	–	[122]
*Galliicola pseudophaeosphaeria*	MFLUCC 14-0524	–	–	–	MG520896	–	–	–	[109]
*Hawksworthiana clematidicola*	MFLUCC 14-0910 ^T^	MG828901	MG829011	–	MG829202	–	MG829120	–	[114]
*Hawksworthiana lonicerae*	MFLUCC 14-0955 ^T^	MG828902	MG829012	–	MG829203	–	MG829121	–	[114]
*Hydeomyces desertipleosporoides*	SQUCC 15260	MK290842	MK290840	–	MK290849	–	MK290844	–	[123]
*Hydeomyces desertipleosporoides*	SQUCC 15259 ^T^	MK290841	MK290839	–	MK290848	–	MK290843	–	[123]
*Hydeomyces pinicola*	GZ-06	MK522506	MK522496	–	MK523386	–	MK522502	–	[124]
*Hydeopsis verrucispora*	SD-2016-5	MK522508	MK522498	–	MK523388	–	MK522504	–	[124]
*Italica achilleae*	MFLUCC 14-0959 ^T^	MG828903	MG829013	–	MG829204	–	MG829122	–	[114]
*Jeremyomyces labinae*	CBS 144617 ^T^	MK442589	–	–	MK442695	–	–	–	[96]
*Juncaceicola italica*	MFLUCC 13-0750	KX500110	KX500107	–	MG520897	–	KX500108	–	[109]
*Juncaceicola luzulae*	MFLUCC 13-0780	KX449529	KX449530	–	MG520898	–	KX449531	–	[125]
*Kwanghwana miscanthi*	FU31017	MK503817	MK503823	–	MT009126	–	MK503829	–	[126]
*Leptosphaeria doliolum*	CBS 505.75 ^T^	JF740205	GU301827	–	GU349069	–	GU296159	–	[127,128]
*Leptospora galii*	KUMCC 15-0521 ^T^	KX599547	KX599548	–	MG520899	–	KX599549	–	[109]
*Leptospora rubella*	CPC 11006	DQ195780	DQ195792	–	–	–	DQ195803	–	[129]
*Leptospora thailandica*	MFLUCC 16-0385 ^T^	KX655559	KX655549	–	KX655564	–	KX655554	–	[130]
*Loratospora luzulae*	MFLUCC 14-0826 ^T^	KT328497	KT328495	–	–	–	KT328496	–	[121]
*Mauginiella scaettae*	CBS 239.58	MH857770	MH869303	–	–	–	–	–	[76]
*Melnikia anthoxanthii*	MFLUCC 14-1010	–	KU848204	–	–	–	KU848205	–	[131]
*Murichromolaenicola chiangraiensis*	MFLUCC 17-1488 ^T^	MN994582	MN994559	–	MN998163	–	MN994605	–	[74]
*Muriphaeosphaeria galatellae*	MFLUCC 15-0769	–	KT438330	–	–	–	KT438332	–	[132]
*Muriphaeosphaeria galatellae*	MFLUCC 14-0614 ^T^	KT438333	KT438329	–	MG520900	–	KT438331	–	[132]
*Neoophiobolus chromolaenae*	MFLUCC 17-1467 ^T^	MN994583	MN994562	–	MN998164	–	MN994606	–	[74]
*Neosetophoma garethjonesii*	MFLUCC 14-0528	–	–	–	KY514402	–	KY501126	–	[133]
*Neosetophoma rosigena*	MFLUCC 17-0768 ^T^	MG828928	MG829037	–	–	–	MG829143	–	[114]
*Neosphaerellopsis thailandica*	CPC 21659 ^T^	KP170652	KP170721	–	KP170678	–	–	–	[118]
*Neostagonospora arrhenather*	MFLUCC 15-0464	KX926417	KX910091	–	MG520901	–	KX950402	–	[134]
*Neostagonospora caricis*	CBS 135092 ^T^	KF251163	KF251667	–	–	–	–	–	[76]
*Neostagonospora phragmitis*	MFLUCC 16-0493	KX926416	KX910090	–	MG520902	–	KX950401	–	[134]
*Neostagonosporella sichuanensis*	MFLUCC 18-1223	MH394690	MH394687	–	MK313854	–	MK296469	–	[58]
*Neostagonosporella sichuanensis*	MFLUCC 18-1228 ^T^	MH368073	MH368079	–	MK313851	–	MH368088	–	[58]
*Neosulcatispora strelitziae*	CPC 25657	KX228253	KX228305	–	–	–	–	–	[112]
*Nodulosphaeria guttulatum*	MFLUCC 15-0069	–	–	–	KY514394	–	KY501115	–	[133]
*Nodulosphaeria multiseptata*	MFLUCC 15-0078	KY496748	KY496728	–	–	–	–	–	[133]
*Nodulosphaeria scabiosae*	MFLUCC 14-1111 ^T^	KU708850	KU708846	–	KU708854	–	KU708842	–	[135]
*Ophiobolopsis italica*	MFLUCC 17-1791 ^T^	MG520939	MG520959	–	MG520903	–	MG520977	–	[109]
*Ophiobolus artemisiae*	MFLUCC 14-1156 ^T^	KT315508	KT315509	–	MG520905	–	MG520979	–	[109]
*Ophiobolus disseminans*	MFLUCC 17-1787	MG520941	MG520961	–	MG520906	–	MG520980	–	[109]
*Ophiobolus ponticus*	MFLUCC 17-2273	MG520943	MG520963	–	MG520908	–	MG520982	–	[109]
*Ophiosimulans tanaceti*	MFLUCC 14-0525	KU738890	KU738891	–	MG520910	–	KU738892	–	[109]
*Ophiosphaerella herpotricha*	KY423	KP690989	–	–	KP691011	–	–	–	[136]
*Ophiosphaerella korrae*	ATCC 56289	KC848509	–	–	KC848515	–	–	–	[136]
*Ophiosphaerella narmari*	ATCC 64688	KC848510	–	–	KC848516	–	–	–	[136]
*Paraleptosphaeria dryadis*	CBS 643.86	JF740213	GU301828	–	GU349009	–	KC584632	–	[127,128]
*Paraleptospora chromolaenae*	MFLUCC 17-1481 ^T^	MN994587	MN994563	–	MN998167	–	MN994609	–	[74]
***Paralloneottiosporina sichuanensis***	**SICAUCC 22-0074 ^T^**	**ON226746**	**ON227102**	**–**	**ON244423**	**–**	**ON227129**	–	This study
***Paralloneottiosporina sichuanensis***	**SICAUCC 22-0075**	**ON226747**	**ON227103**	**–**	**ON244424**	**–**	**ON227130**	–	This study
*Paraloratospora camporesii*	MFLU 18-0915 ^T^	MN756639	MN756637	–	–	–	MN756635	–	[113]
*Paraophiobolus arundinis*	MFLUCC 17-1789 ^T^	MG520945	MG520965	–	MG520912	–	MG520984	–	[109]
*Paraophiobolus plantaginis*	MFLUCC 17-0245 ^T^	KY797641	KY815010	–	–	–	KY815012	–	[109]
*Paraphoma chrysanthemicola*	CBS 522.66	KF251166	KF251670	–	KF253124	–	–	–	[116]
*Paraphoma radicina*	CBS 111.79	KF251172	KF251676	–	KF253130	–	–	–	[116]
*Parastagonospora italica*	MFLUCC 13-0377 ^T^	KU058714	KU058724	–	MG520915	–	MG520985	–	[109,137]
*Parastagonospora minima*	MFLUCC 13-0376	KU058713	KU058723	–	MG520916	–	MG520986	–	[109,137]
*Parastagonosporella fallopiae*	CCTU 1151.1	MH460544	MH460546	–	MH460550	–	–	–	[138]
*Parastagonosporella fallopiae*	CBS 135981 ^T^	MH460543	MH460545	–	MH460549	–	–	–	[138]
*Phaeopoacea festucae*	MFLUCC 17-0056	KY824766	KY824767	–	–	–	KY824769	–	[134]
*Phaeoseptoriella zeae*	CBS 144614 ^T^	MK442611	MK442547	–	MK442702	–	–	–	[96]
*Phaeosphaeria chiangraina*	MFLUCC 13-0231 ^T^	KM434270	KM434280	–	KM434298	–	KM434289	–	[57]
*Phaeosphaeria oryzae*	CBS 110110 ^T^	KF251186	KF251689	–	–	–	GQ387530	–	[139]
*Phaeosphaeria pleurospora*	CBS 460.84	AF439498	–	–	–	–	–	–	[140]
*Phaeosphaeriopsis glaucopunctata*	MFLUCC 13-0265	KJ522473	KJ522477	–	MG520918	–	KJ522481	–	[109,141]
*Phaeosphaeriopsis triseptata*	MFLUCC 13-0271	KJ522475	KJ522479	–	MG520919	–	KJ522484	–	[109,141]
*Phaeosphaeriopsis yuccae*	MFLUCC 16-0558	KY554482	KY554481	–	MG520920	–	KY554480	–	[109]
*Piniphoma wesendahlina*	CBS 145032 ^T^	MK442615	MK442551	–	MK442706	–	–	–	[96]
*Poaceicola arundinis*	MFLUCC 15-0702 ^T^	KU058716	KU058726	–	MG520921	–	MG520988	–	[109]
*Poaceicola italica*	MFLUCC 13-0267	KX926421	KX910094	–	MG520924	–	KX950409	–	[109,134]
*Populocrescentia ammophilae*	MFLUCC 17-0665 ^T^	MG828949	MG829059	–	MG829231	–	MG829164	–	[114]
*Populocrescentia forlicesenensis*	MFLUCC 14-0651 ^T^	KT306948	KT306952	–	MG520925	–	KT306955	–	[121]
*Populocrescentia rosae*	TASM 6125 ^T^	–	MG829060	–	MG829232	–	MG829165	–	[114]
*Pseudoophiobolus mathieui*	MFLUCC 17-1784	MG520949	MG520969	–	MG520928	–	MG520991	–	[109]
*Pseudoophiobolus rosae*	MFLUCC 17-1786 ^T^	MG520952	MG520972	–	MG520930	–	MG520993	–	[109]
*Pseudoophiobolus urticicola*	KUMCC 17-0168 ^T^	MG520955	MG520975	–	MG520933	–	MG520996	–	[109]
*Pseudoophiosphaerella huishuiensis*	HS-13	MK522509	MK522499	–	MK523389	–	MK522505	–	[124]
*Pseudophaeosphaeria rubi*	MFLUCC 14-0259 ^T^	KX765298	KX765299	–	MG520934	–	KX765300	–	[130]
*Sclerostagonospora ericae*	CPC 25927 ^T^	KX228268	KX228319	–	KX228375	–	–	–	[112]
*Scolicosporium minkeviciusii*	MFLUCC 12-0089	–	KF366382	–	–	–	KF366383	–	[142]
*Septoriella phragmitis*	CPC 24118 ^T^	KR873251	KR873279	–	–	–	–	–	[143]
*Setomelanomma holmii*	CBS 110217	KT389542	GU301871	–	GU349028	–	GU296196	–	[127,144]
*Setophoma terrestris*	CBS 335.29	KF251246	KF251749	–	KF253196	–	–	–	[116]
*Stagonospora neglecta*	CBS 343.86	AJ496630	–	–	–	–	–	–	[145]
*Sulcispora supratumida*	MFLUCC 14-0995 ^T^	KP271443	KP271444	–	MH665366	–	KP271445	–	[146]
*Tintelnotia destructans*	CBS 127737 ^T^	KY090652	KY090664	–	–	–	KY090698	–	[147]
*Tintelnotia opuntiae*	CBS 376.91 ^T^	KY090651	GU238123	–	–	–	GU238226	–	[147,148]
*Vagicola arundinis*	MFLUCC 15-0027 ^T^	KY706139	KY706129	–	MG520936	–	KY706134	–	[109]
*Vittaliana mangrovei*	NFCCI 4251 ^T^	MG767311	MG767312	–	MG767314	–	MG767313	–	[149]
*Vrystaatia aloeicola*	CBS 135107	KF251278	KF251781	–	–	–	–	–	[116]
*Wingfieldomyces cyperi*	CBS 141450 ^T^	KX228286	KX228337	–	MK540163	–	–	–	[150]
*Wojnowicia italica*	MFLUCC 13-0447 ^T^	KX342923	KX430001	–	KX430003	–	KX430002	–	[130]
*Wojnowicia rosicola*	MFLUCC 15-0128 ^T^	MG828979	MG829091	–	–	–	MG829191	–	[114]
*Wojnowiciella eucalypti*	CBS 139904 ^T^	KR476741	KR476774	–	–	–	–	–	[76]
*Xenophoma puncteliae*	CBS 128022	–	JQ238619	–	KP170686	–	–	–	[118,151]
*Xenoseptoria neosaccardoi*	CBS 120.43	KF251280	KF251783	–	KF253227	–	–	–	[116]
*Xenoseptoria neosaccardoi*	CBS 128665	KF251281	KF251784	–	KF253228	–	–	–	[116]
*Yunnanensis phragmitis*	MFLUCC 17-1361 ^T^	MF684869	MF684865	–	–	–	MF684864	–	[152]
*Yunnanensis phragmitis*	MFLUCC 17-0315	MF684862	MF684863	–	MF683624	–	MF684867	–	[152]
*Biatriospora marina*	CY 1228	–	GQ925848	–	GU479848	–	GQ925835	GU479823	[153]
*Biatriospora peruviensis*	CCF 4485	–	LN626683	–	LN626671	–	LN626677	LN626665	[154]
*Neooccultibambusa chiangraiensis*	MFLUCC 12-0559 ^T^	–	KU764699	–	–	–	KU712458	–	[155]
*Neoroussoella bambusae*	MFLUCC 11-0124	–	KJ474839	–	KJ474848	–	–	KJ474856	[156]
*Occultibambusa aquatica*	MFLUCC 11-0006	–	KX698110	–	–	–	KX698112	–	[130]
*Occultibambusa bambusae*	MFLUCC 11-0394	–	KU863113	–	KU940194	–	KU872117	KU940171	[16]
*Occultibambusa bambusae*	MFLUCC 13-0855 ^T^	–	KU863112	–	KU940193	–	KU872116	KU940170	[16]
*Occultibambusa chiangraiensis*	MFLUCC 16-0380 ^T^	–	KX655546	–	–	–	KX655551	KX655566	[130]
*Occultibambusa fusispora*	MFLUCC 11-0127 ^T^	–	KU863114	–	KU940195	–	–	KU940172	[16]
*Occultibambusa jonesii*	GZCC 16-0117 ^T^	–	KY628322	–	KY814756	–	KY628324	KY814758	[157]
*Occultibambusa kunmingensis*	HKAS 102151 ^T^	–	MN913733	–	MT954407	–	MT864342	MT878453	[61]
*Occultibambusa maolanensis*	GZCC 16-0116	–	KY628323	–	KY814757	–	KY628325	KY814759	[157]
*Occultibambusa pustula*	MFLUCC 11-0502	–	KU863115	–	–	–	KU872118	–	[16]
*Paradictyoarthrinium diffractum*	MFLUCC 13-0466	–	KP744498	–	–	–	KP753960	KX437764	[105,158]
*Paradictyoarthrinium tectonicola*	MFLUCC 13-0465 ^T^	–	KP744500	–	–	–	KP753961	KX437763	[105,158]
*Roussoella hysterioides*	HH 26988	–	AB524622	–	AB539115	–	AB524481	AB539102	[127]
*Roussoella nitidula*	MFLUCC 11-0182	–	KJ474843	–	KJ474852	–	–	KJ474859	[156]
*Roussoella nitidula*	MFLUCC 11-0634	–	KJ474842	–	KJ474851	–	–	KJ474858	[156]
*Roussoella pustulans*	KT 1709	–	AB524623	–	AB539116	–	AB524482	AB539103	[1,127]
*Seriascoma bambusae*	KUMCC 21-0021	–	MZ329035	–	MZ325468	–	MZ329031	MZ325470	[159]
*Seriascoma didymospora*	MFLUCC 11-0179 ^T^	–	KU863116	–	KU940196	–	–	KU940173	[16]
*Seriascoma didymospora*	MFLUCC 11-0194	–	KU863117	–	KU940197	–	–	KU940174	[16]
*Seriascoma yunnanense*	MFLU 19-0690 ^T^	–	NG_068303	–	MN381858	–	MN174694	MN210324	[44]
***Seriascoma yunnanense***	**SICAUCC 22-0059**	**–**	**ON226771**	**–**	**ON567182**	**–**	**ON227356**	**ON567183**	This study
*Torula herbarum*	CBS 111855	–	KF443386	–	KF443403	–	KF443391	KF443396	[160]
*Westerdykella ornata*	CBS 379.55	–	GU301880	–	GU349021	–	GU296208	GU371803	[127]

Notes: superscript ^T^ represents ex-type or ex-epitype isolates. “–” means that the sequence is missing, unavailable or unused. New sequences are listed in bold. Abbreviation: AP: Culture Collection of A. Pintos; ATCC: American Type Culture Collection, U.S.A.; BRIP: Queensland Plant Pathology Herbarium, Brisbane, Australia; CBS: Culture Collection of the Westerdijk Fungal Biodiversity Institute, Utrecht, The Netherlands; CFCC: China Forestry Culture Collection Center, Beijing, China; CGMCC: China General Microbiological Culture Collection Center, Institute of Microbiology, Chinese Academy of Sciences, Beijing, China; CPC: Culture Collection of P.W. Crous; DAOM: Plant Research Institute, Department of Agriculture (Mycology), Ottawa, Canada; GZCC: Guizhou Academy of Agricultural Sciences Culture Collection, Guizhou, China; IMI: Culture Collection of CABI Europe UK Centre, Egham, UK; JHB: Culture Collection of H.B. Jiang; KUMCC: Kunming Institute of Botany Culture Collection, Yunnan, China; LC: Working collection of Lei Cai, housed at the Institute of Microbiology, Chinese Academy of Sciences, Beijing, China; MFLU: Herbarium of Mae Fah Luang University, Chiang Rai, Thailand; MFLUCC: Mae Fah Luang University Culture Collection, Chiang Rai, Thailand; NCYUCC: National Chiayi University Culture Collection, Chiayi, Taiwan; SICAUCC: Sichuan Agricultural University Culture Collection, Sichuan, China; URM: Culture Collection of the Universidade Federal de Pernambuco, Brazil.
